# Bats, Primates, and the Evolutionary Origins and Diversification of Mammalian Gammaherpesviruses

**DOI:** 10.1128/mBio.01425-16

**Published:** 2016-11-08

**Authors:** Marina Escalera-Zamudio, Edith Rojas-Anaya, Sergios-Orestis Kolokotronis, Blanca Taboada, Elizabeth Loza-Rubio, Maria L. Méndez-Ojeda, Carlos F. Arias, Nikolaus Osterrieder, Alex D. Greenwood

**Affiliations:** a Department of Wildlife Diseases, Leibniz Institute for Zoo and Wildlife Research (IZW), Berlin, Germany; b Centro Nacional de Investigación Disciplinaria en Microbiología Animal—INIFAP, Mexico City, Mexico; c Department of Epidemiology and Biostatistics, School of Public Health, SUNY Downstate Medical Center, Brooklyn, New York, USA; d Departamento de Genética del Desarrollo y Fisiología Molecular, Instituto de Biotecnología, Universidad Nacional Autónoma de México, Cuernavaca, Morelos, Mexico; e Facultad de Medicina Veterinaria y Zootecnia, Universidad Veracruzana, Veracruz, Mexico, and Institut für Virologie, Robert von Ostertag-Haus-Zentrum für Infektionsmedizin, Department of Veterinary Medicine, Freie Universität Berlin, Berlin, Germany; f Department of Veterinary Medicine, Freie Universität Berlin, Berlin, Germany

## Abstract

Gammaherpesviruses (γHVs) are generally considered host specific and to have codiverged with their hosts over millions of years. This tenet is challenged here by broad-scale phylogenetic analysis of two viral genes using the largest sample of mammalian γHVs to date, integrating for the first time bat γHV sequences available from public repositories and newly generated viral sequences from two vampire bat species (*Desmodus rotundus* and *Diphylla ecaudata*). Bat and primate viruses frequently represented deep branches within the supported phylogenies and clustered among viruses from distantly related mammalian taxa. Following evolutionary scenario testing, we determined the number of host-switching and cospeciation events. Cross-species transmissions have occurred much more frequently than previously estimated, and most of the transmissions were attributable to bats and primates. We conclude that the evolution of the *Gammaherpesvirinae* subfamily has been driven by both cross-species transmissions and subsequent cospeciation within specific viral lineages and that the bat and primate orders may have potentially acted as superspreaders to other mammalian taxa throughout evolutionary history.

## IMPORTANCE

It has long been believed that herpesviruses have coevolved with their hosts and are species specific. Nevertheless, a global evolutionary analysis of bat viruses in the context of other mammalian viruses, which could put this widely accepted view to the test, had not been undertaken until now. We present two main findings that may challenge the current view of γHV evolution: multiple host-switching events were observed at a higher rate than previously appreciated, and bats and primates harbor a large diversity of γHVs which may have led to increased cross-species transmissions from these taxa to other mammals.

## INTRODUCTION

The *Herpesviridae* are a large group of DNA viruses within the order *Herpesvirales* that infect many vertebrate host species (1). It is widely accepted that herpesviruses have codiverged with their hosts for millions of years and that they are generally species specific (1). Nonetheless, ancient spillover events that led to viral divergence and adaptation to new hosts have been detected for some viral groups (2, 3). The *Herpesviridae* family comprises three subfamilies, the *Alpha-*, *Beta*-, and *Gammaherpesvirinae*, with the latter mainly including lymphotropic viruses that can result in lymphoproliferative disease, such as the Epstein-Barr virus (EBV) or Kaposi sarcoma-associated herpesvirus (KSHV) (1). Gammaherpesviruses (γHVs) establish latent life-long infections but generally cause disease only in naive or immunosuppressed individuals (1). The four genera within the *Gammaherpesvirinae* subfamily are *Percavirus*, *Macavirus*, *Lymphocryptovirus*, and *Rhadinovirus* (1). Percaviruses are considered to have originated in perissodactyls (mainly equids) and carnivores (felids and mustelids), macaviruses in artiodactyls, and lymphocryptoviruses in primates. Only the *Rhadinovirus* genus displays a broader distribution among different mammalian orders, represented by a polyphyletic assemblage within the γHV phylogeny (2, 4).

Although several γHVs have been detected in different bat species, there has been no thorough examination of the evolutionary history of these viruses (5–12). Therefore, the evolution of bat γHVs in the context of other mammalian viruses remains largely unexplored, while the lack of γHVs described in Neotropical bats has biased the overall representation of bat γHV diversity. To test the hypothesis of host-restricted virus coevolution within the *Gammaherpesvirinae* subfamily, we explored the evolutionary dynamics of the bat and other mammalian γHVs. We included new bat viral sequences generated from two vampire bat species that occupy a wide geographical range on the American continent: *Desmodus rotundus* (the common vampire bat) and *Diphylla ecaudata* (the hairy-legged vampire bat). Our evolutionary analysis did not support the hypothesis of a strict cospeciation (CS) scenario and further revealed that viral cross-species transmission occurred most frequently from bats and primates to other taxa, with subsequent viral adaptation and coevolution within the recipient mammalian hosts.

## RESULTS

### Detection of vampire bat γHVs by serology and PCR.

Fourteen of 21 *D. rotundus* and 2 of 3 *D. ecaudata* bat individuals from Veracruz (Soledad Doblado locality) were positive for herpesviruses, determined by a panherpesvirus PCR targeting a 150- to 200-bp region in the viral DNA polymerase gene (*dpol*) (Table 1; see also Table S1 in the supplemental material). In contrast, only two of six *D. rotundus* bats from Morelos and none of the bats from Estado de Mexico were positive. However, such low prevalence may be a result of the limited sampling size. Many of the viral sequences identified from vampire bats matched by BLASTN a previously described γHV from *Pteropus giganteus* (PgHV-5) (host, Indian flying fox; GenBank accession number AGW27609.1) (5) with a sequence identity of >90% (Table 1). Surprisingly, the viral sequences detected in the samples from the *D. ecaudata* individuals SD16 and SD12 matched those of a *Macaca fuscata* rhadinovirus isolate, 12E2 (host, Japanese macaque; GenBank accession number JN885137.1), and a *Babyrousa babyrussa* rhadinovirus 1 isolate (host, golden babirusa hog; GenBank accession number AY177146.2). BLASTX consistently revealed that many of the viral coding sequences were most similar to the PgHV-5 DNA polymerase protein (Pol). However, the viral sequences from *D. rotundus* individual MOR4 and *D. ecaudata* SD16 were most similar to the bovine herpesvirus 4 (BoHV-4) Pol (host, cattle; GenBank accession number AIA82756.1). The sequence from *D. ecaudata* SD12 was highly similar to that of the *Myotis ricketti* herpesvirus 2 Pol (host, Rickett’s big-footed bat; GenBank accession number JN692430.1) and the sequence from *D. rotundus* individual SD3 to the phascolarctid herpesvirus 1 Pol (host, koala; GenBank accession number AEX15649). An additional PCR targeting 500 bp of the γHV glycoprotein B gene (*gB*) (2) yielded products for two (*D. ecaudata* SD12 and *D. rotundus* MOR4) of the 32 samples tested (Table 1). *D. ecaudata* SD12 matched the *Macaca fuscata* rhadinovirus isolate 12E2 with a 70% nucleotide sequence identity, while *D. rotundus* MOR4 yielded a moderate (66% nucleotide identity) match to *Saimiriine herpesvirus 2* (host, common squirrel monkey; GenBank accession number AAA46164). BLASTX showed comparable results, supporting similarity to the primate γHV gB protein in both cases (Table 1). To determine whether the *gB* and *dpol* sequences in these two samples belonged to the same virus, we attempted to amplify a syntenic block containing *gB* and *dpol* by long-range PCR (LR-PCR) (2) but failed to obtain any products.

**TABLE 1:** Bat samples PCR positive for γHVs

**PCR target, sample**^a^	**BLASTN best hit**^a^	**E-value**	**% identity**	**Length (bp)**	**BLASTX best hit**^a^	**E-value**	**% identity**	**Length (aa)**
*dpol^c^*								
DrMOR2	PgHV-5 *dpol*	4E-52	98	126	PgHV-5 Pol	2E-20	98	42
DrMOR4	PgHV-5 *dpol*	8E-10	74	306^b^	BoHV-4 Pol	3E-11	59	102^b^
DeSD16	MfusRHV 12E2 *dpol*	1E-14	74	140	BoHV-4 Pol	1E-29	56	46
DeSD12	BbabRHV-1 *dpol*	1E-20	73	495^b^	MrGHV-2 Pol	5E-21	77	165^b^
DrSD1	PgHV-5 *dpol*	2E-63	97	149	PgHV-5 Pol	1E-25	98	49
DrSD3	PgHV-5 *dpol*	6E-06	70	144	PhaHV-1 Pol	1E-08	57	46
DrSD5	PgHV-5 *dpol*	7E-55	99	128	PgHV-5 Pol	4E-21	100	42
DrSD6	PgHV-5 *dpol*	7E-49	98	120	PgHV-5 Pol	4E-19	100	39
DrSD9	PgHV-5 *dpol*	1E-65	98	151	PgHV-5 Pol	2E-27	100	50
DrSD10	PgHV-5 *dpol*	4E-65	97	152	PgHV-5 Pol	2E-26	98	49
DrSD11	PgHV-5 *dpol*	5E-64	98	148	PgHV-5 Pol	1E-26	100	49
DrSD17	PgHV-5 *dpol*	5E-51	98	124	PgHV-5 Pol	1E-20	100	41
DrSD18	PgHV-5 *dpol*	7E-62	98	144	PgHV-5 Pol	6E-26	100	48
DrSD19	PgHV-5 *dpol*	2E-62	97	149	PgHV-5 Pol	3E-22	93	46
DrSD22	PgHV-5 *dpol*	4E-65	93	178	PgHV-5 Pol	6E-24	92	51
DrSD23	PgHV-5 *dpol*	3E-47	97	119	PgHV-5 Pol	7E-18	97	39
DrSD24	PgHV-5 *dpol*	4E-59	99	134	PgHV-5 Pol	2E-22	100	44
DrSD25	PgHV-5 *dpol*	5E-45	99	108	PgHV-5 Pol	1E-15	100	35
*gB^d^*								
DeSD12	MfusRHV 12E2 *gB*	1E-41	70	420	PtroRHV-2 gB	1E-59	73	140
DrMOR4	HVS2 *gB*	6E-17	66	489	MfusRHV JM12 gB	1E-62	64	163

a Dr, *Desmodus rotundus*; De, *Diphylla ecaudata*; PgHV-5, *Pteropus giganteus* herpesvirus 5; MrGHV-2, *Myotis ricketti* herpesvirus 2; MfusRHV, *Macaca fuscata* rhadinovirus; BbabRHV-1, *Babyrousa babyrussa* rhadinovirus 1; PtroRHV-2, *Pan troglodytes* rhadinovirus 2; PhaHV-1, phascolarctid herpesvirus 1; HVS, *Saimiriine herpesvirus*.

b Sequence was extended by HTS.

c Total positives from the bat individuals tested, 18/32.

d Total positives from the bat individuals tested, 2/32.

Given the distant genetic relatedness of some of the vampire bat viruses to BoHV-4, we used a BoHV-4-diagnostic enzyme-linked immunosorbent assay (ELISA) kit to determine the antigenic similarities and seroprevalences of γHVs within the bat populations studied. Serology showed that the sera of four bat individuals from the Soledad Doblado locality (two of which, *D. ecaudata* SD12 and SD16, were also positive by the *dpol* PCR) cross-reacted with BoHV-4, suggesting an antigenic relatedness between the vampire bat γHVs and BoHV-4 (see Fig. S1 in the supplemental material). There was no cross-reactivity observed to the other vampire bat or to the equid serum controls tested (data not shown).

### Confirmation of vampire bat γHV sequences by high-throughput sequencing.

To provide additional evidence for the presence of γHVs in vampire bats, high-throughput sequencing (HTS) was performed on five selected samples that were previously determined to be PCR positive for γHVs. Approximately 400 million raw reads with a size distribution of 100 to 300 bp were obtained (48 to 92 million reads per library) and were sequentially filtered to obtain verifiable high-quality reads (see Table S2 in the supplemental material). For *D. rotundus* MOR4, 32 reads matched 15 different γHV genes, with 3 reads matching the *gB* gene and 1 read matching the *dpol* gene (see Table S3). For *D. rotundus* individual SD2, 5 reads were assigned to 4 different viral genes, while for *D. rotundus* SD3, 10 reads were assigned to 7 different genes, although no reads matched *gB* or *dpol*. In the case of *D. ecaudata* SD12, 31 reads were assigned to more than 15 different γHV genes, with 2 reads matching *dpol* and 1 read matching *gB*. Finally, for *D. ecaudata* SD16, 33 reads were assigned to more than 15 viral genes, with two of them matching *dpol* but none matching *gB* (see Table S3). In all cases, the viral sequences matched mostly bat, bovid, and primate γHVs*.* Given that the vast majority of sequences obtained were expected to match the host genome, contig assembly was not performed with the raw data. However, assembly from the filtered reads generated extended contigs for three samples (*D. rotundus* MOR4 and *D. ecaudata* SD12 and SD16), yielding sequences of up to 735 bp matching, again, bovid and primate γHVs (see Table S4). Such results supported the conclusion that vampire bats carry bovine and primate γHV-like viruses.

### Wide distribution of bat γHV viruses among mammalian γHV lineages.

**FIG 1:**
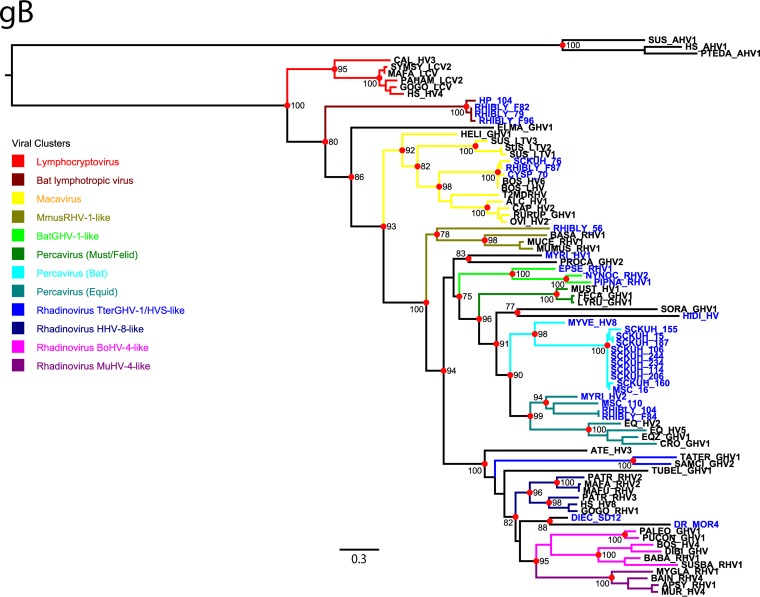
The phylogeny of gammaherpesviruses based on a 564-residue-long alignment of the glycoprotein B (gB) sequence. Maximum-likelihood tree estimated from 81 mammalian γHV sequences, including 30 viral sequences from 14 different bat species. The tree is color coded according to the major γHV clusters, while bat viral sequences are highlighted in blue. The tree was rooted with alphaherpesvirus sequences. Branch support values are shown for nodes with support values of >70% according to the Shimodaira-Hasegawa-like approximate-likelihood ratio test (SH-like–aLRT), represented by red circles. The full names for the viral isolates and their original hosts are available in Table S5 in the supplemental material. The scale bar denotes amino acid substitutions per site.

Ten main viral lineages have been described for the *Gammaherpesvirinae* subfamily: *Lymphocryptovirus*, *Macavirus*, *Mus musculus* rhadinovirus 1 (MmusRHV-1)-like, bat gammaherpesvirus 1 (BatGHV-1)-like, *Percavirus*, *Rhadinovirus Tapirus terrestris* gammaherpesvirus 1 (TterGHV-1)-like, *Rhadinovirus Herpesvirus saimiri* (HVS), *Rhadinovirus Human herpesvirus* 8 (HHV-8)-like, *Rhadinovirus* murid herpesvirus 4 (MuHV-4)-like, and *Rhadinovirus* BoHV-4 (2, 4). For the gB tree, all previously described lineages were detected, showing a comparable resolution to previously published topologies (Fig. 1) (2, 4). However, in addition to the BatGHV-1-like group, bat γHVs were found to be widely distributed among 6 mammalian viral lineages previously thought to be order specific (2, 4). The most important differences observed between our gB tree and the previously published phylogenetic trees (2, 4) were as follows: (i) the identification of a new bat virus cluster (designated here “bat lymphotropic viruses”) diverging from the basal lymphocryptoviruses; (ii) the presence of bat viral sequences forming a sister group to the bovine lymphotropic viruses within the *Macavirus* lineage; (iii) a bat-derived viral sequence basal to the MmusRHV-1-like viruses; (iv) the *Percavirus* lineage splitting into three subclusters isolated from mustelids/felids, bats, and equids; and (v) the grouping of vampire bat viral sequences between the *Rhadinovirus* HHV-8-like and the *Rhadinovirus* BoHV-4-like groups. The viral sequences from bats often represented deep branches within the tree, such as for the bat lymphotropic virus group and the MmusRHV-1-like and BatGHV-1-like clusters (Fig. 1). We further compared the gB topology obtained to three different plausible evolutionary scenarios within a maximum-likelihood (ML) inference framework: (i) strict virus-host cospeciation, (ii) a strict bat origin for all γHVs, and (iii) monophyly for bat γHVs. Our results revealed that the alternative tree topologies were not supported by the data (SH test, *P* ≤ 0.01; expected-likelihood weight [ELW] of best ML tree, posterior probability [PP] = 1.0), indicating that the phylogenetic pattern we observed most likely reflects the evolutionary history of γHVs.

Given the short lengths of many of the Pol sequences and the few variable sites for phylogenetic inference by standard approaches, we used the Evolutionary Placement Algorithm (EPA), in which the short bat viral sequences were treated as short reads and assigned to nodes within a reference tree based on their likelihood weight ratios (LWR). If a given sequence has a single high value for the LWR (see Fig. S2 in the supplemental material, red circles), then its placement within a particular branch or node of the tree is supported. If a sequence has many possibilities for placement, then it can have many low LWR values. The overall confidence for sequence placement is expressed by the entropy value of each sequence, where low entropy indicates good confidence for placement. In the absence of a threshold, we considered a placement to be confident only for sequences with a single LWR value of ≥0.4 within a branch or node or with cumulative LWR values of ≥0.3 within a same cluster (Table 2). Although many of the bat sequences could not be placed on the tree with high confidence, an overall pattern similar to that of the gB tree was observed, including a basal position for some of the bat viral sequences and a wide distribution among different mammalian viral lineages (see Fig. S2). Sequences were assigned with confidence to the following viral clusters: *Lymphocryptovirus*, *Macavirus*, MmusRHV-1-like, BatGHV-1-like, *Percavirus*, *Rhadinovirus* HVS, and *Rhadinovirus* HHV-8-like groups (Table 2). The resulting topology is publicly available as an interactive project on the Interactive Tree of Life (iToL) version 3 webserver (http://itol.embl.de/tree/21616595883251465841813).

**TABLE 2:** Phylogenetic placement of the Pol bat viral sequences on the reference tree

Bat γHV^a^	LWR	Cluster
NYNOC_RHV1	0.60	*Lymphocryptovirus*
PTGIG_HV5	0.75	*Lymphocryptovirus*
CYSP_M102	0.94	*Macavirus*
RHIBLY_F99	0.89	*Macavirus*
SCKUH_84	0.94	*Macavirus*
HP_110	0.64	*Percavirus*
RHIBLY_F84	0.94	*Percavirus*
SCKUH_M121	0.39	*Percavirus^b^*
SCKUH_15	0.32	*Percavirus^b^*
SCKUH_239	0.39	*Percavirus^b^*
SCKUH_16	0.38	*Percavirus^b^*
PLAUR_RHV1	0.56	BatGHV-1-like
MYNA_RHV1	0.34	BatGHV-1-like^b^
PIPI_RHV1	0.50	BatGHV-1-like
SCKUH_146	0.44	MmusRHV-1-like
SCKUH_M185	0.44	MmusRHV-1-like
DIEC_SD12	0.41	*Rhadinovirus* HHV-8-like
HL_HN1	0.69	*Rhadinovirus* HVS

a Names of viruses represented by abbreviations here are available in Table S5 in the supplemental material.

b Sequence was not assigned to a particular branch, due to a low LWR, but had a cumulative LWR supporting its placement within the given viral cluster.

### Multiple bat and primate transmissions to other mammals.

**FIG 2:**
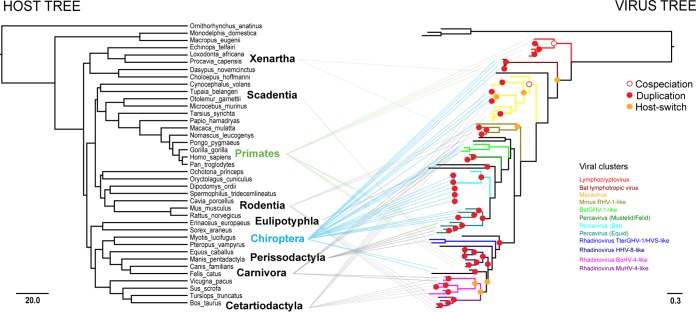
Tanglegram of the host-virus coevolution within the *Gammaherpesvirinae* subfamily. Higher host taxonomic levels are denoted in bold font. The virus phylogeny is represented by the gB tree. The gray lines indicate the connections between particular mammalian orders and viral lineages. The names and connecting lines of the two main groups where the most host-switching events were detected are shown in blue (bats) and green (primates). The estimated cospeciation (open circles), duplication (red circles), and host-switching events (yellow circles) obtained by cophylogeny analysis are shown on the virus tree. The scale bars indicate millions of years before present for the host tree (left) and amino acid substitutions per site for the virus tree (right).

The virus and host phylogenies were compared to estimate the numbers of primary and secondary host switches (HS) and cospeciation (CS) events. The resulting tanglegram revealed multiple HS within the gB phylogeny, most of them attributable to the order Chiroptera (Fig. 2). Ten primary HS occurring at the order level were detected, 3 of which were attributed to bat γHVs (bat lymphotropic viruses to *Elephas maximus* gammaherpesvirus 1 [ELMA_GHV1], BatGHV-1-like to mustelid/felid *Percavirus*, and bat to equid *Percavirus*), and 2 were attributed to primates (lymphocryptovirus to bat lymphotropic viruses and *Rhadinovirus* HHV-8-like to the *Rhadinovirus* MuHV-4-like and BoHV-4-like groups). The remaining 5 HS were single events attributable to different taxonomic groups: ELMA_GHV1 to *Macavirus* (Afrotheria to Artiodactyla), *Macavirus* to the MmusRHV-1-like group (Artiodactyla to Rodentia), MmusRHV-1-like to *Percavirus* (Rodentia to multiple hosts), Percaviruses to the *Rhadinovirus* supercluster (multiple hosts to multiple hosts), and *Tupaia belangeri* gammaherpesvirus 1 (TUBEL_GHV1) to the HHV-8-like rhadinoviruses (Scadentia to Primates). Secondary HS events occurring at a species level revealed a total of 6 HS, 3 of which involved bat viruses; these included bovine lymphotropic herpesviruses and the bat viruses *Scotophilus kuhlii* γHV 11HZ76 (SCKUH_76), *Rhinolophus blythi* γHV 13YF87 (RHIBLY_F87), and *Cynopterus sphinx* γHV 13HN70 (CYSP_70) within the *Macavirus* group, the *Rhinolophus blythi* γHV 13HN56 (RHIBLY_56) isolate within the MmusRHV-1-like viruses, and *Myotis ricketti* herpesvirus 1 (MYRI_HV1) next to the *Procavia capensis* gammaherpesvirus 2 (PROCA_GHV2). Other secondary HS events included *Mustelid herpesvirus 1* (MUST_HV1) and felid γHVs (*Lynx rufus* gammaherpesvirus 1 [LYRU_GHV1] and *Felis catus* gammaherpesvirus 1 [FECA_GHV1]) within the percaviruses, *Tapirus terrestris* gammaherpesvirus 1 (TATER_GHV1) and *Saimiri sciureus* gammaherpesvirus 2 (SAMCI_GHV2) within the TterGHV-1 group and the felid rhadinoviruses (*Puma concolor* gammaherpesvirus 1 [PUCON_GHV1] and *Panthera leo* gammaherpesvirus 1 [PALEO_GHV1]) within the *Rhadinovirus* BoHV-4 group. Cospeciation was detected within the *Lymphocryptovirus*, *Macavirus*, MmusRHV-1-like, BatGHV-1-like, felid, bat, and equid *Percavirus*, *Rhadinovirus* HHV-8-like, *Rhadinovirus* MuHV-4-like, and *Rhadinovirus* BoHV-4-like groups, yielding a total of 10 CS events. In agreement with our results, the optimal solution obtained by the cophylogeny analysis revealed that duplications and host-switching events outnumber the cospeciation events, while this reconciliation was statistically supported (*P* < 0.05). Further supporting our previous observations, most duplication/HS events were detected within the chiropterans, with 34 duplications and 5 HS, followed by 15 duplications in primates, 10 duplications and 2 HS in artiodactyls, 4 duplications and 1 HS in carnivores, and finally, 3 duplications in both rodents and perissodactyls. Within the parsimony framework of minor costs, only 2 cospeciation events were detected (Fig. 2).

### Limited homology between viral and host proteins.

It is possible that some of the accessory γHV open reading frames (ORFs) known to have cellular homologs would share a significant sequence identity to the host protein counterparts, if cospeciation had occurred (13). Thus, we examined the amino acid sequence similarity between the viral and host FLICE-inhibitory-like protein (FLIP), B-cell lymphoma-2 apoptosis (BCL-2) mediator protein, and OX-2 membrane glycoprotein. No significant identity to mammalian proteins was detected for the viral BCL-2 (vBCL-2) and vOX-2 proteins. However, vFLIP resembled mammalian CASP8 and FADD-like apoptosis regulator protein (cFLAR) death effector domain 1 and 2 (DED1/DED2; amino acids [aa] 1 to 172). Our results revealed that while most of the γHV FLIP proteins shared significant identity to cFLARs of diverse mammalian species (mostly rodents, bats, and primates), only vFLIP from MYVE_HV8 shared identity with the cFLAR protein of the *Myotis* genus, suggesting cospeciation (see Table S6 in the supplemental material). Nonetheless, such results should be interpreted with caution, as cFLAR is highly conserved among all mammals (>70% identity in amino acids) and only shares a weak similarity to vFLIP (<50% identity in amino acids).

## DISCUSSION

The genetic and antigenic characterization of the vampire bat viral sequences revealed that these viruses are distantly related to other bat, bovid, and primate γHVs. However, the genetic distance among sequences suggests that the vampire bat viruses are divergent and may have become established in the vampire bat population long ago. We detected most of the γHV sequences in the spleen, which is consistent with both viral replication tissue tropism and latency occurring in germinal center B cells, as has been described for a number of other mammalian γHVs (14). Based on preliminary analyses of the *D. rotundus* genome (M. Lisandra Zepeda Mendoza, Zijun Xiong, Marina Escalera-Zamudio, Anne Kathrine Runge, Julien Thézé, Daniel Streicker, Hannah K. Frank, Elizabeth Loza-Rubio, Shengmao Liu, Oliver A. Ryder, Jose Alfredo Samaniego Castruita, Aris Katzourakis, Blanca Taboada, Ulrike Löber, Oliver G. Pybus, Yang Li, Edith Rojas-Anaya, Kristine Bohmann, Aldo Carmona Baez, Carlos F. Arias, Shiping Liu, Alex D. Greenwood, Mads Frost Bertelsen, Nicole E. White, Mike Bunce, Guojie Zhang, Thomas Sicheritz-Pontén, M. Thomas P. Gilbert, unpublished data), there is no evidence for integration of γHVs into the vampire bat genome. Therefore, the novel virus sequences described in this work are unlikely to emanate from endogenized γHVs. The relatively small amount of HTS reads obtained suggests that the vampire bat viral sequences stem from latent viruses. However, consistent with the possibility of viral reactivation from splenic B cells, a higher concentration of reads was detected in two vampire bat samples that were also γHV positive by PCR and serology (*D. ecaudata* SD12 and SD16). Vampire bats are the only mammals that feed exclusively on the blood of other animals, and at least in the case of *D. rotundus*, they have a preference for domestic swine and bovids. Vampire bats have been selectively feeding on the blood of cattle since their introduction in the Americas, as they represent an easily accessible food source (15). Thus, some of the BoHV-4-related γHVs in vampire bats might have been introduced into these bat species as a consequence of dietary specialization. However, our results do not support that vampire bats are outliers among other bats in terms of harboring more γHVs, as most of the bat species where γHVs have been detected are insectivorous (*Eptesicus serotinus*, *Hipposideros diadema*, *Hipposideros larvatus*, *Hipposideros pomona*, *Miniopterus schreibersii*, *Myotis nattereri*, *Myotis velifer*, *Nyctalus noctula*, *Pipistrellus nathusii*, *Pipistrellus pipistrellus*, *Plecotus auritus*, *Rhinolophus blythi*, and *Scotophilus kuhlii*), frugivorous (*Cynopterus sphinx*, *Ptenochirus jagori*, and *Pteropus giganteus*), and in one case, piscivorous (*Myotis ricketti*) (16). Therefore, feeding ecology may not be a critical factor in cross-species transmission. It has been recently suggested that the process of host switching is strongly influenced by the opportunity to encounter a new host presented to the parasite and by the compatibility of a parasite for colonizing a new host, given that the host selective pressure may not be strong enough to eliminate the parasite (17). Moreover, parasites can persist for extended periods in suboptimal hosts until reaching a new niche through a stepping-stone process, circulating in different hosts that can be divergent from each other but in relative physical proximity (17). Thus, we speculate that bat-specific traits, such as flight, large population sizes, and a wide geographical range, might have been important in enabling or enhancing γHV spillover from bats to other taxa.

We used the largest collection of mammalian γHV sequences to date, representing all lineages of the *Gammaherpesvirinae* subfamily within 34 different taxa and including the γHV sequences available from 19 different bat species. Because of the lack of viral sequences for many mammalian orders and a sampling bias in primates, ungulates, and rodents, it is likely that the diversity and evolution of γHVs is still not fully represented with the current data. This could explain the long branches observed between many viral isolates within different viral phylogenetic clusters. However, only further sampling within a larger diversity of hosts will help determine the full scale of viral diversity, and this will likely reveal additional cross-species transmission events not detected here. Although the availability of different γHV groups and genes within GenBank is limited, we used a comprehensive data set to include the largest possible number of viruses within the widest range of hosts, by employing the best-represented viral ORFs (*dpol* and *gB*) that are least likely to have reached mutational saturation over the long evolutionary time scale examined (data not shown). Although phylogenetic analyses have been carried out in previous studies (2, 4), reduced data sets of 12 to 45 viral sequences were used, with bat γHVs being underrepresented. Bat herpesvirus discovery and characterization has relied mostly on sequences obtained by PCR, which often represent short amplicons because of DNA quality and sample limitation issues (2, 8, 12). Using short sequences for phylogenetic analysis has caveats, but analyzing different viral genes independently, including sufficient full-length sequences, and using alternative phylogenetic approaches, such as the placement of shorter sequences on a reference tree, can increase confidence in phylogenetic inference.

Our results revealed that the overall phylogenetic pattern for γHVs observed from two independent viral genes is not congruent with a strict virus-host codivergence scenario. Our data strongly support cross-species transmissions within viral clusters that were thought to be order specific (*Macavirus*, MmusRHV-1-like, and *Percavirus*). Moreover, several primate and bat viral lineages represent deep branches within the γHV phylogeny, such as the *Lymphocryptovirus* group that is basal to the bat lymphotropic viruses, and may thus represent the oldest viral lineages. Our results further suggest that primates and bats may carry the highest diversity of γHVs, while the close phylogenetic relationship between some of the bat and primate viral groups provides evidence for ancient spillover events, as has been observed for other herpesviruses (3, 18). Furthermore, the similarity between viruses present in distantly related bat species suggests that some bat γHVs are likely to be very old and to have emerged shortly after the divergence of chiropterans at least 60 million years ago (MYA) (19, 20). However, these viruses may have maintained the ability to jump between different mammalian species, as observed for the bat γHVs that are closely related to the BoHV-6 isolates within the *Macavirus* clade (12). An origin for γHV emergence was estimated at approximately 64 MYA by extrapolating the divergence dates of swine and ruminant hosts to the viruses within the *Macavirus* genus (2, 4). Although this assumption may be valid for the viruses found within artiodactyl hosts, it is likely that γHVs in general are much older, possibly coinciding with the origin of placental mammals at least 84 MYA (21). Nonetheless, given the limited length of many of the γHV sequences, estimating a chronology for the diversification of the overall viral group and for more-shallow clusters would likely yield inaccurate dates (22).

The evolution of specific γHV lineages not being compatible with a strict virus-host cospeciation had been previously noted (2). The ratio of cospeciation versus duplication and host-switching events, which we detected both manually and by cophylogeny analysis, suggests that although cospeciation might have occurred for particular lineages, it was often preceded by duplication and/or a host-switching event. Host switching was also detected within viral groups previously thought to be order specific. Together, these observations suggest that cross-species transmission followed by lineage-specific cospeciation have been the main evolutionary drivers within the *Gammaherpesvirinae* subfamily. Furthermore, alternative topology testing revealed that strict cospeciation is not supported by the data, congruent with a polyphyletic origin for most γHVs. A strict bat origin for γHVs was also not supported, suggesting that many species have played a role in the sequential spread of γHVs throughout evolutionary history (8, 11, 12). Hence, we propose that the *Gammaherpesvirinae* subfamily has evolved by many interspecies transfers, with specific host codivergence playing a role in γHV evolution only after adaptation to a new host. Our data indicate that chiropterans and primates may have played an important role in γHV transmission, as has been observed for other viral groups (23). However, future analyses using other viral genomic regions and a greater sampling of viral diversity should help to clarify the full extent and timing of viral cross-species transmission at different evolutionary timescales.

## MATERIALS AND METHODS

### Nucleic acid extraction and PCR.

Bat sample collection was approved by the Internal Committee for Ethics and Animal Welfare (approval no. 2012-09-05) and was carried out in compliance with Mexican regulations (collection permit NUM/SGPA/DGVS/03173/14; export certificate SAGARPA 241111524599811488A467371). Twenty-nine *D. rotundus* and three *D. ecaudata* bats (*n* = 32) were captured using mist nets in three different localities in Mexico (San Pablo, Tlaltizapán Morelos, Mexico; Soledad Doblado, Veracruz, Mexico; and La Cabecera, Estado de Mexico, Mexico) (see Table S1 in the supplemental material). Because sampling was dependent on bat seasonality, we were only able to obtain a limited number of individuals for each species and from each locality. Spleen tissue from 32 sacrificed animals was used for nucleic acid extraction (QIAamp MinElute virus spin kit; Qiagen) as previously described. A universal nested PCR for the detection of herpesviruses targeting a short fragment (150 to 200 bp) of the viral DNA polymerase gene (*dpol*) was used to screen each bat tissue sample (2, 24). Further PCRs using virus-specific primers targeting a 500-bp region of the γHV glycoprotein B gene (*gB*) and to cover the genetic distance between *gB* and *dpol* using long-distance PCR (LD-PCR) were carried out as previously described (2). PCR products were visualized on 1.5% (wt/vol) agarose gels stained with Midori green (Nippon Genetics) and Sanger sequenced using BigDye version 3 chemistry on an ABI 3730xl DNA analyzer (Life Technologies) at LCG Genomics (Berlin, Germany). To determine sequence identity, sequences were analyzed by BLASTN and by BLASTX (https://blast.ncbi.nlm.nih.gov/Blast.cgi).

### Serology.

Fresh blood from each bat was obtained using the Microvette CB 300-µl system (Sarstedt) and centrifuged for 5 min at 10,000 × *g* at 20°C for serum separation. All sera were stored at −20°C for further use. Given the lack of standardized enzyme-linked immunosorbent assay (ELISA) kits for wildlife, a commercial kit available for bovine herpesvirus 4 (BoHV-4) diagnostics in cattle (BIO K 263; Bio-X Diagnostics, Belgium) was used. This assay uses whole virus for detection, and thus, cross-reactivity with related γHVs is likely. Additionally, it uses a protein G-horseradish peroxidase (HRP) conjugate that is able to detect immunoglobulins from most mammalian species, including bats. ELISA was performed following the manufacturers’ instructions, using 5 serial dilutions of each bat’s serum (1:10, 1:25, 1:50, 1:100, and 1:200) and including the diluted negative and positive cattle serum controls provided with the kit. An optimal serum dilution of 1:50 was standardized for the bat samples, while a cutoff value of 30% compared to the positive control (value = ΔOD sample × 100/ΔOD positive-control serum, where OD is optical density) was used to determine positive sera, following the manufacturer’s instructions. Further external controls were added to test for cross-reactivity against other mammalian alphaherpesviruses and γHVs. For this purpose, 3 equid sera determined to be positive for different γHVs by PCR and one serum positive for equine herpesvirus 1 by ELISA were tested under the conditions described above. Given the limited amount of bat samples available, a single test with duplicate reactions was carried out.

### High-throughput DNA sequencing.

DNA samples from five bat individuals positive for γHVs by PCR (*D. rotundus* MOR4, *D. ecaudata* SD16, *D. ecaudata* SD12, *D. rotundus* SD2, and *D. rotundus* SD3) were used to prepare double-indexed Illumina libraries (25). Prior viral enrichment steps were not possible given the field collection conditions. Individual genomic libraries were pooled for 2 × 150-bp paired-end sequencing on the Illumina NextSeq 500 platform with the NextSeq version 2 kit on high-output mode at the Berlin Center for Genomics in Biodiversity Research (BeGenDiv). Sequence reads were quality filtered and adapters removed, followed by host DNA filtering and viral taxonomic assignment (26). High-quality reads were filtered to remove bacterial, human, and chiropteran sequences by mapping with SMALT version 0.7.6 (http://sanger.ac.uk/resources/software/smalt) under a stringency of 50 to 70% against custom-built genomic libraries retrieved from the Reference genomic sequence (refseq_genomic) NCBI database (http://www.ncbi.nlm.nih.gov/refseq/about/) and against the *D. rotundus* genomic data (Zepeda-Mendoza et al., unpublished data). Viral assignment was performed using BLASTX version 2.2.29 (http://blast.ncbi.nlm.nih.gov/Blast.cgi) against the GenBank nonredundant protein database and mapped with SMALT against a custom-built herpesviral database under a stringency of 60%. The γHV-matching reads were further selected by reciprocal BLASTX analysis using the following criteria: length of ≥100 bp, pairwise identity of >50%, E-value of <10^−6^, and independent hits to two different γHV proteins or at least two different regions of the same protein. Although this last step may significantly reduce the final number of reads, it is important in order to obtain verifiable as opposed to sporadic hits. It has been proposed that for metagenomic approaches using wildlife samples, only reads above ≥150 bp in coding sequences and yielding identity to different viral protein targets can be considered accurate for pathogen identification (27). From the filtered reads, contigs were assembled to obtain longer sequences using SAMtools version 1.3.1 (28) and SMALT to map against the consensus sequence at a stringency of 60%.

### Sequence alignment and estimation of variable sites.

For gB, the 92 available protein-coding sequences from viruses isolated from diverse mammalian species (including most of the bat and reference viruses) were retrieved from the GenBank nonredundant nucleotide database as of May 2016. After collapsing identical sequences and pruning to eliminate redundancy and short/low-quality sequences, a total number of 81 sequences were retained for the analysis (see Table S5 and Data Set S1 in the supplemental material). From the 81 sequences used, only 21 corresponded to full-length protein sequences, while the remaining 60 were partial sequences with an average length of 290 to 163 aa. Saturation within the nucleotide sequences was estimated to discard the possible effects of long-branch attraction (LBA) (data not shown). Translated amino acid sequences were aligned through sequential profile alignments for divergent sequences using MUSCLE, as implemented in SeaView (29, 30). The alignment was manually edited to remove highly divergent regions, resulting in a final length of 564 aa, comparable to the data sets used in previous studies (636 aa) (4). For Pol, the same procedure as for gB was followed, resulting in an alignment of 97 OTUs with a length of 894 aa, comparable to data sets used in previous studies (909 aa) (see Table S5 and Data Set S2) (4). From the sequences characterized in this work, only two DR-γHV sequences, with a length of >100 aa (*D. rotundus* MOR4 and *D. ecaudata* SD12), were included in both gB and Pol alignments for phylogenetic analysis. In order to assess the number of variable sites attributed to the bat sequences, the original gB and Pol data sets were modified to shortened versions trimmed to the average length of the bat sequences. For gB, an alignment of 189 aa (minimum length of 140 aa) was obtained, while for Pol, an alignment of 74 aa (minimum length of 55 aa) was retrieved, excluding outgroup sequences.

### Phylogenetic analysis.

The best-fit amino acid substitution model for gB was identified using jModelTest2 (31) (LG and empirical residue frequencies +F, with among-site rate heterogeneity modeled by the Γ distribution with four rate categories) (32, 33), while phylogenetic analysis was performed under maximum likelihood (ML) using RAxML version 8.2.8 (34). Ten searches starting from stepwise-addition maximum-parsimony trees were run, while node robustness was assessed by the Shimodaira-Hasegawa [SH]-like (35) approximate-likelihood ratio test (aLRT). Given the short length of the bat viral sequences and the reduced number of variable sites for Pol, we used the Evolutionary Placement Algorithm (EPA) for the assignment of sequence fragments to a reference tree using the maximum-likelihood optimality criterion in RAxML (34, 36) with the aforementioned model parameters (LG+Γ_4_+F). All viral sequences of <250 amino acids were treated as short reads and assigned within a reference sequence alignment and ML tree based on their likelihood weight ratios (LWR). To obtain the reference tree, bat viral sequences were pruned from the original full-length alignment, leaving only the 60 longer reference viral sequences (4). The phylogenetic mapping of the short sequences was visualized using the Interactive Tree of Life (iToL) version 3 online tool (http://itol.embl.de) (37).

### Alternative evolutionary scenario testing.

Phylogenetic testing was performed for three different gB evolutionary scenarios: (i) strict host-virus cospeciation, (ii) a strict bat origin for all γHVs (bat sequences are monophyletic at a basal position on the tree), and (iii) a single origin for bat γHVs (bat sequences are monophyletic within the BatGHV-1-like viral cluster). The different evolutionary scenarios were tested in RAxML using (i) the Shimodaira-Hasegawa (SH) test (35) for contrasting the best ML tree and alternative topologies and (ii) the expected-likelihood weight (ELW) procedure (38) to establish a confidence tree set using 100 bootstrap samples.

### Comparison of the host-virus phylogeny.

For the host tree, the UCSC 100-way vertebrate genome phylogenetic tree based on the 100-way BLAST search to obtain orthologs of the opsin gene *ONP5* (neuropsin) (http://hgdownload.cse.ucsc.edu/goldenpath/hg19/phyloP100way) (39) was manually edited to display an even representation of 39 species belonging to the euarchontoglires and laurasiatherian mammalian superorders. For the host-virus phylogeny comparison, the gB tree was contrasted with the host tree using the tanglegram algorithm for rooted phylogenies implemented in Dendroscope version 3 (40). As bat viruses within the gB tree represent 38% of all sequences used, to minimize the effects of sampling bias (e.g., a larger number of viral sequences available for particular taxonomic groups) in the interpretation of the results, only the number of viral lineages represented for each mammalian order, and not the number of viral sequences available for each taxonomic group, was taken into account.

### Cophylogeny analysis.

The numbers of primary and secondary host-switching (HS) events versus cospeciation (CS) events within the gB tree were manually counted. Primary host-switching events were defined on an ordinal level as a viral lineage derived from a host (order) diverging from another viral lineage from another host (order). Secondary HS events were defined on a species level as a viral sublineage derived from a host (species) grouping basally or next to another viral sublineage from a different host (species). CS events were observed as order- or species-specific viral lineages that demonstrate a strict viral host codivergence. Under these criteria, only nodes with a support value of ≥80% were considered. Furthermore, Jane4 (41) was used to test for significant congruence between the virus and host trees, searching for evidence for coevolution. Jane4 is suitable for assessing unbalanced numbers of hosts and parasites and multihost parasitism. It uses a heuristic approach based on maximum parsimony to search for tree reconciliation solutions between associated phylogenies by minimizing the overall costs given by individual evolutionary events between host and parasite, as follows: (i) cospeciation, (ii) duplication (a parasite speciates but remains on the same host), (iii) host switching (a parasite speciates and shifts onto a different host), (iv) loss (a host speciates but the parasite remains only on one of the new hosts), and (v) failure to diverge (a host speciates and the parasite remains on both old and new host) (41). The cost regimes tested were as follows: default cost settings within the range of [0, 3]. Generation times of 10, 50, and 100 were run with population sizes set to 10, 30, and 50 with 100 replicates. The optimal solutions were examined, and the probability of each cophylogeny having arisen by chance was calculated. The lower-cost optimal solution was compared within the corresponding simulated empirical distribution.

### Homology analysis of accessory ORFs.

Accessory γHV ORFs (vFLIP, vBCL-2, and vOX-2) with known homology to mammalian protein counterparts (13) were retrieved through manual searches in GenBank (http://www.ncbi.nlm.nih.gov/genbank/) and within the 18 fully annotated γHV genomes available in the Reference genomic sequence (refseq_genomes) NCBI database (http://www.ncbi.nlm.nih.gov/refseq/about/). Sequences for vFLIP were obtained for 9 viruses (ATE_HV3, BOS_HV4, EQ_HV2, EQ_HV5, FECA_GHV1, HS_HV8, MAFU_RHV, MYVE_HV8, and SAM_HV2), and sequences for vBCL-2 were retrieved for 14 viruses (ALC_HV1, ATE_HV3, BOS_HV4, BOS_HV6, EQ_HV2, EQ_HV5, FECA_GHV1, HS_HV8, MUR_HV4, MYVE_HV8, OVI_HV2, SAM_HV2, SUS_LTV2, and SUS_LTV3), while a single vOX-2 sequence was retrieved for one virus (MYVE_HV8). Complete virus names are available in Table S5 in the supplemental material. In order to determine significant global sequence identities within the viral and host proteins, the retrieved protein sequences were analyzed with PSI-BLAST using default parameters and a 0.005 PSI-BLAST statistical significance threshold (25).

### Accession number(s).

GenBank accession numbers for the viral sequences used in the phylogenetic analysis are listed in Table S5 in the supplemental material. Vampire bat viral sequences were deposited in GenBank under the following accession numbers: *Desmodus rotundus* MOR4 Pol (KU942401), *Diphylla ecaudata* SD16 Pol (KU942402), *Diphylla ecaudata* SD12 Pol (KU942403), *Desmodus rotundus* MOR4 gB (KU942404), and *Diphylla ecaudata* SD12 gB (KU942405). Given the short length (≤200 bp) of some of the sequences determined in this study, not all vampire bat viral sequences could be deposited in GenBank, but these are available upon request. HTS data are available from the Dryad Digital Repository (http://dx.doi.org/10.5061/dryad.sg0k6). The HTS reads were deposited on the NCBI Sequence Read Archive (SRA) under BioProject number PRJNA348455.

## SUPPLEMENTAL MATERIAL

Figure S1  Seroprevalence of BoHV-4-related viruses in vampire bats. An ELISA was performed on 32 bat sera using an optimized dilution of 1:50, also diluting the negative and positive controls included in the kit. A cutoff value of 30% compared to the value for the positive control was used for positive samples (value = ΔOD sample × 100/ΔOD positive-control serum). Error bars on each circle represent the standard deviation obtained from the two reaction values for each sample. Closed circles indicate sera from individuals negative by *dpol* PCR, while open circles indicate PCR-positive individuals. Download Figure S1, TIF file, 1.4 MB

Figure S2  Placement of the short-length Pol bat viral sequences within the reference tree. The reference tree for Pol was inferred using 60 mammalian viruses, while the Evolutionary Placement Algorithm (EPA) was used to place the short bat viral sequences within the standard tree. Placements are shown by the red circles showing values for the likelihood weight ratios (LWR) and thus represent the confidence. If a given sequence has a single high LWR value (large red circles) rather than several LWR values (small red circles), then its placement within a particular branch or node is supported. Only single LWR values of ≥0.4 or cumulative LWR values of ≥0.3 within a same cluster were considered. Download Figure S2, TIF file, 1.3 MB

Table S1  Bat individuals and localities sampled. Table S1, DOCX file, 0.01 MB

Table S2  Read counts for each library. Table S2, DOCX file, 0.01 MB

Table S3  BLASTX search results for the verifiable reads assigned to γHVs. Table S3, DOCX file, 0.03 MB

Table S4  BLASTX search results for assembled contigs from the filtered γHVs reads. Table S4, DOCX file, 0.01 MB

Table S5  Accession numbers for the DNA polymerase (*dpol*) and glycoprotein B (*gB*) sequences from γHVs used in this study. Table S5, DOCX file, 0.03 MB

Table S6  Significant identities determined by PSI-BLAST between the amino acid sequences of the viral and host FLIP/FLAR proteins. Table S6, DOCX file, 0.1 MB

Data Set S1  gB amino acid alignment. Download Data Set S1, TXT file, 0.04 MB

Data Set S2  Pol amino acid alignment. Download Data Set S2, TXT file, 0.1 MB
