# Supplementary figures and images for: Bats, Primates, and the Evolutionary Origins and Diversification of Mammalian Gammaherpesviruses
Source: mBio. 2016 Nov 8;7(6):e01425-16. doi: 10.1128/mBio.01425-16 (PMC5101351; doi:10.1128/mBio.01425-16)

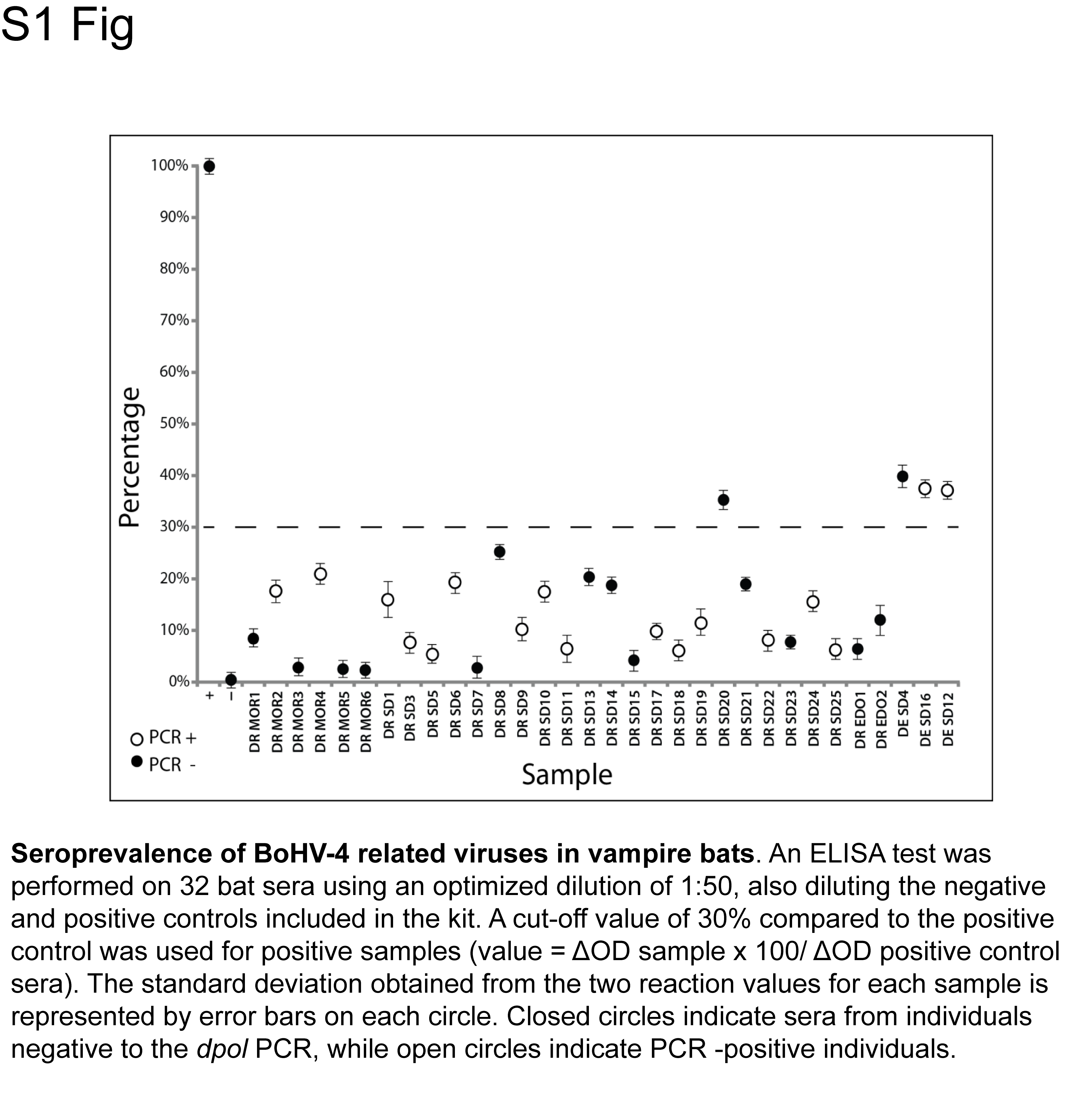

Supplement: Figure S1 — Seroprevalence of BoHV-4-related viruses in vampire bats. An ELISA was performed on 32 bat sera using an optimized dilution of 1:50, also diluting the negative and positive controls included in the kit. A cutoff value of 30% compared to the value for the positive control was used for positive samples (value = ΔOD sample × 100/ΔOD positive-control serum). Error bars on each circle represent the standard deviation obtained from the two reaction values for each sample. Closed circles indicate sera from individuals negative by dpol PCR, while open circles indicate PCR-positive individuals. Download [file mbo005163037sf1.tif]

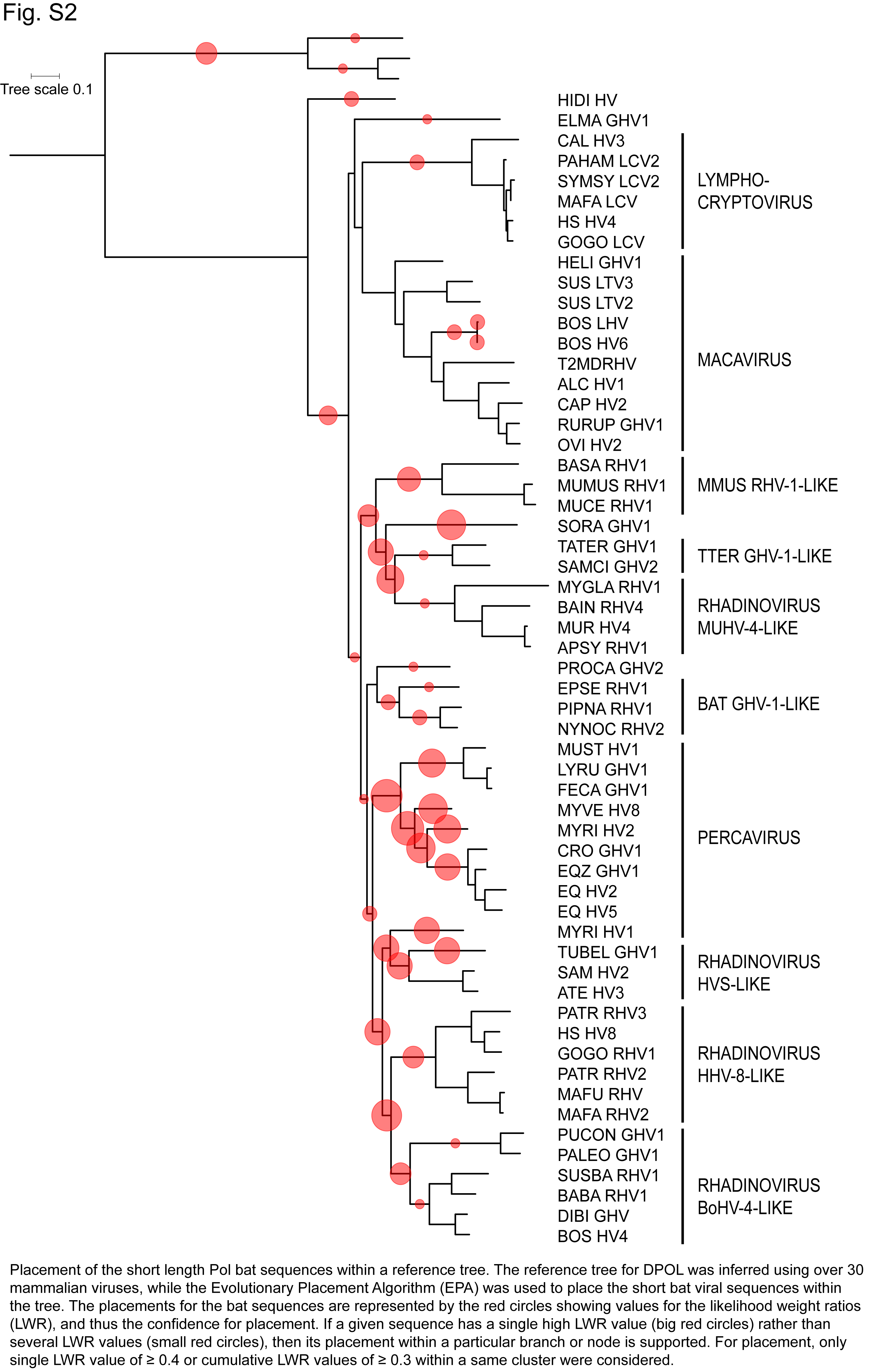

Supplement: Figure S2 — Placement of the short-length Pol bat viral sequences within the reference tree. The reference tree for Pol was inferred using 60 mammalian viruses, while the Evolutionary Placement Algorithm (EPA) was used to place the short bat viral sequences within the standard tree. Placements are shown by the red circles showing values for the likelihood weight ratios (LWR) and thus represent the confidence. If a given sequence has a single high LWR value (large red circles) rather than several LWR values (small red circles), then its placement within a particular branch or node is supported. Only single LWR values of ≥0.4 or cumulative LWR values of ≥0.3 within a same cluster were considered. Download [file mbo005163037sf2.tif]
