# Supplementary material for: Bats, Primates, and the Evolutionary Origins and Diversification of Mammalian Gammaherpesviruses
Source: mBio. 2016 Nov 8;7(6):e01425-16. doi: 10.1128/mBio.01425-16 (PMC5101351; doi:10.1128/mBio.01425-16)
Supplement: Table S1 — Bat individuals and localities sampled. [file mbo005163037st1.docx]

**TABLE S1** Bat individuals and localities sampled

| **Species/Locality**^1,2,3^ | **No. Individuals** |
| --- | --- |
| *Desmodus rotundus MOR* | 6 |
| *Desmodus rotundus SD* | 21 |
| *Diphylla ecaudata SD* | 3 |
| *Desmodus rotundus EDO* | 2 |
| **Total:** | **32** |

^1^ MOR= San Pablo, Tlaltizapan Morelos, Mexico. N 18º52'23.81'' W 99º06'02.81''

^2^ SD= Soledad Doblado, Veracruz, Mexico. N 18º59'34.03'' W 96º27'28.21''

^3^ EDO= La Cabecera, Estado de Mexico, Mexico. N 18º53'45.66'' W 100º11'03.1397''
