# Supplementary material for: Bats, Primates, and the Evolutionary Origins and Diversification of Mammalian Gammaherpesviruses
Source: mBio. 2016 Nov 8;7(6):e01425-16. doi: 10.1128/mBio.01425-16 (PMC5101351; doi:10.1128/mBio.01425-16)
Supplement: Table S2 — Read counts for each library. [file mbo005163037st2.docx]

| **Sample** | **TOTAL R1/R2** | **Quality filtering** | **% Reads lost** | **Host filtering** | **% Reads lost** | **Assigned to γHV** | **%** | **Unassigned reads** | **%** |
| --- | --- | --- | --- | --- | --- | --- | --- | --- | --- |
| *Desmodus rotundus* MOR4 | 48144060 | 40853878 | 15 | 34473712 | 71 | 1610 | 0,003 | 6378556 | 13 |
| *Diphylla ecaudata* SD16 | 89804751 | 73390091 | 18 | 56756995 | 63 | 1024 | 0,001 | 16632072 | 18 |
| *Diphylla ecaudata* SD12 | 91057852 | 72924294 | 19 | 37585199 | 41 | 4987 | 0,005 | 35334108 | 38 |
| *Desmodus rotundus* SD2 | 82075595 | 68100951 | 17 | 32637730 | 39 | 2832 | 0,003 | 35460389 | 43 |
| *Desmodus rotundus* SD3 | 62600899 | 51738563 | 17 | 43327364 | 69 | 1884 | 0,003 | 8409315 | 13 |
| NC | 376 |  |  |  |  |  |  |  |  |
| Undetermined | 32039928 |  |  |  |  |  |  |  |  |
| TOTAL READS: | 405723461 |  |  |  |  |  |  |  |  |

**TABLE S2** Read counts for each library
