# Supplementary material for: Bats, Primates, and the Evolutionary Origins and Diversification of Mammalian Gammaherpesviruses
Source: mBio. 2016 Nov 8;7(6):e01425-16. doi: 10.1128/mBio.01425-16 (PMC5101351; doi:10.1128/mBio.01425-16)
Supplement: Table S3 — BLASTX search results for the verifiable reads assigned to γHVs. [file mbo005163037st3.docx]

**TABLE S3** Blastx results for the verifiable reads assigned to γHVs

| **SAMPLE (READS)** | **BLAST ID** | **E-value** | **% ID** | **Lenght (aa)** | **Lenght (nt)** |
| --- | --- | --- | --- | --- | --- |
| ***Desmodus rotundus herpesvirus MOR4*** |  |  |  |  |  |
| NB500882:12:HHG75BGXX:1:13209:23740:16382 | major capsid protein [Equid herpesvirus 5] | 9E-21 | 54 | 74 | 222 |
| NB500882:12:HHG75BGXX:1:22201:12120:18647 | glycoprotein B [Macaca fascicularis rhadinovirus 2] | 3E-33 | 70 | 87 | 261 |
| NB500882:12:HHG75BGXX:2:22108:2326:2738 | glycoprotein B [Macaca fascicularis rhadinovirus 2] | 3E-33 | 70 | 87 | 261 |
| NB500882:12:HHG75BGXX:2:12210:19254:9803 | gB protein [Saimiriine herpesvirus 2] | 9E-14 | 48 | 71 | 213 |
| NB500882:12:HHG75BGXX:2:23304:18473:2222 | protein UL79 [Equid herpesvirus 2] | 9E-14 | 68 | 44 | 132 |
| NB500882:12:HHG75BGXX:2:21309:21838:17012 | capsid protein [Bovine herpesvirus 4] | 6E-17 | 82 | 45 | 135 |
| NB500882:12:HHG75BGXX:3:12505:24781:14778 | DNA packaging tegument protein UL25 [Myotis gammaherpesvirus 8] | 5E-20 | 58 | 92 | 276 |
| NB500882:12:HHG75BGXX:3:21602:11408:9293 | transport protein [Ateline herpesvirus 3] | 8E-27 | 58 | 84 | 252 |
| NB500882:12:HHG75BGXX:3:13610:25044:15490 | DNA polymerase [Semnopithecus entellus lymphocryptovirus 1] | 2E-14 | 58 | 55 | 165 |
| NB500882:12:HHG75BGXX:3:23605:24372:17020 | ribonucleotide reductase subunit 2 [Myotis gammaherpesvirus 8] | 2E-18 | 91 | 43 | 129 |
| NB500882:12:HHG75BGXX:4:13607:5761:2024 | capsid triplex subunit 1 [Equid herpesvirus 2] | 5E-19 | 67 | 58 | 174 |
| NB500882:12:HHG75BGXX:1:13305:24058:12395 | envelope glycoprotein M [Murid herpesvirus 4] | 3E-19 | 79 | 48 | 144 |
| NB500882:12:HHG75BGXX:1:21201:3671:13580 | hypothetical protein [Saimiriine herpesvirus 2] | 2E-11 | 42 | 74 | 222 |
| NB500882:12:HHG75BGXX:1:21311:19328:3717 | hypothetical protein [Bovine herpesvirus 4] | 2E-25 | 70 | 66 | 198 |
| NB500882:12:HHG75BGXX:1:22302:24868:2298 | ORF17 [Macaca nemestrina rhadinovirus 2] | 1E-27 | 59 | 92 | 276 |
| NB500882:12:HHG75BGXX:1:23111:7197:17361 | unnamed protein product [Saimiriine herpesvirus 2] | 1E-15 | 57 | 65 | 195 |
| NB500882:12:HHG75BGXX:1:23307:6560:10744 | single-stranded DNA-binding protein MDBP [Bovine herpesvirus 4] | 3E-20 | 68 | 60 | 180 |
| NB500882:12:HHG75BGXX:2:11302:2104:13403 | major capsid protein [Bovine herpesvirus 4] | 2E-38 | 71 | 91 | 273 |
| NB500882:12:HHG75BGXX:2:23108:20592:16995 | ORF18 [Felis catus gammaherpesvirus 1] | 4E-28 | 62 | 77 | 231 |
| NB500882:12:HHG75BGXX:2:22209:2199:18861 | major capsid protein [Bovine herpesvirus 4] | 8E-21 | 76 | 50 | 150 |
| NB500882:12:HHG75BGXX:3:12608:11406:3952 | helicase [Saimiriine herpesvirus 2] | 1E-25 | 57 | 99 | 297 |
| NB500882:12:HHG75BGXX:3:11607:25803:13292 | unnamed protein product [Saimiriine herpesvirus 2] | 3E-12 | 41 | 86 | 258 |
| NB500882:12:HHG75BGXX:3:11605:12721:6124 | DNA packaging tegument protein UL25 [Myotis gammaherpesvirus 8] | 1E-16 | 62 | 60 | 180 |
| NB500882:12:HHG75BGXX:3:12609:15134:16066 | uracil-DNA glycosylase [Myotis gammaherpesvirus 8] | 2E-14 | 69 | 59 | 177 |
| NB500882:12:HHG75BGXX:4:22407:19740:10315 | hypothetical protein [Saimiriine herpesvirus 2] | 8E-13 | 59 | 54 | 162 |
| NB500882:12:HHG75BGXX:4:12503:8287:7323 | unnamed protein product [Saimiriine herpesvirus 2] | 3E-12 | 41 | 86 | 258 |
| NB500882:12:HHG75BGXX:4:22505:9765:11333 | major capsid protein [Saimiriine herpesvirus 2] | 1E-33 | 84 | 70 | 210 |
| NB500882:12:HHG75BGXX:4:13512:8558:9530 | hypothetical protein MmrVgp80 [Macacine herpesvirus 5] | 7E-16 | 62 | 63 | 189 |
| NB500882:12:HHG75BGXX:4:22408:23369:1149 | ORF 6 [Human herpesvirus 8 type M] | 1E-16 | 65 | 68 | 204 |
| NB500882:12:HHG75BGXX:4:11503:22528:19859 | unknown [Bovine herpesvirus 4] | 2E-17 | 68 | 57 | 171 |
| NB500882:12:HHG75BGXX:4:13412:18523:8525 | unnamed protein product [Saimiriine herpesvirus 2] | 5E-21 | 79 | 48 | 144 |
| NB500882:12:HHG75BGXX:1:21210:16516:2797 | packaging protein [Rhesus monkey rhadinovirus H26-95] | 2E-13 | 73 | 41 | 123 |
|  |  |  |  |  |  |
| ***Desmodus rotundus herpesvirus SD2*** |  |  |  |  |  |
| NB500882:12:HHG75BGXX:1:12304:25021:17089 | single-stranded DNA-binding protein MDBP [Bovine herpesvirus 4] | 2E-30 | 61 | 85 | 255 |
| NB500882:12:HHG75BGXX:1:23108:10702:13962 | single-stranded DNA-binding protein MDBP [Bovine herpesvirus 4] | 6E-13 | 71 | 45 | 135 |
| NB500882:12:HHG75BGXX:1:11101:16159:4594 | major capsid protein [Saimiriine herpesvirus 2] | 1E-23 | 72 | 65 | 195 |
| NB500882:12:HHG75BGXX:1:13310:17313:1968 | orf 39 [Ateline herpesvirus 3] | 2E-31 | 62 | 90 | 270 |
| NB500882:12:HHG75BGXX:1:12202:10720:6153 | glycoprotein M [Babyrousa babyrussa rhadinovirus 1] | 6E-13 | 73 | 40 | 120 |
|  |  |  |  |  |  |
| ***Desmodus rotundus herpesvirus SD3*** |  |  |  |  |  |
| NB500882:12:HHG75BGXX:3:23503:6809:17506 | transport protein [Bovine herpesvirus 4] | 8E-25 | 65 | 72 | 216 |
| NB500882:12:HHG75BGXX:3:12412:15351:15873 | ribonucleotide reductase large subunit [Bovine herpesvirus 4] | 9E-17 | 67 | 57 | 171 |
| NB500882:12:HHG75BGXX:1:12211:17617:1538 | ribonucleotide reductase large subunit [Bovine herpesvirus 4] | 1E-16 | 65 | 52 | 156 |
| NB500882:12:HHG75BGXX:1:12106:1916:2429 | ORF60 [Macaca nemestrina rhadinovirus 2] | 1E-27 | 71 | 68 | 204 |
| NB500882:12:HHG75BGXX:1:11203:25376:15638 | orf 29B [Ateline herpesvirus 3] | 6E-25 | 70 | 66 | 198 |
| NB500882:12:HHG75BGXX:2:22205:8436:17427 | single-stranded DNA-binding protein MDBP [Bovine herpesvirus 4] | 2E-20 | 73 | 55 | 165 |
| NB500882:12:HHG75BGXX:4:13602:6212:9529 | major ssDNA binding protein [Ateline herpesvirus 3] | 5E-25 | 60 | 84 | 252 |
| NB500882:12:HHG75BGXX:4:23405:26136:12832 | hypothetical protein [Saimiriine herpesvirus 2] | 2E-20 | 62 | 61 | 183 |
| NB500882:12:HHG75BGXX:4:22506:13287:2823 | helicase [Bovine herpesvirus 4] | 2E-16 | 76 | 45 | 135 |
|  |  |  |  |  |  |
| ***Diphylla ecaudata herpesvirus SD12*** |  |  |  |  |  |
| NB500882:12:HH75BXX:2:22307:11831:19597 | tegument protein [Bovine herpesvirus 4] | 1E-11 | 41 | 78 | 234 |
| NB500882:12:HH75BXX:1:11301:14581:8444 | tegument protein UL7 [Myotis gammaherpesvirus 8] | 5E-24 | 53 | 92 | 276 |
| NB500882:12:HH75BXX:1:22104:24859:19621 | helicase-primase primase subunit [Equid herpesvirus 5] | 3E-22 | 51 | 91 | 273 |
| NB500882:12:HH75BXX:1:11205:14776:2145 | F18 [Felis catus gammaherpesvirus 1] | 6E-17 | 48 | 86 | 258 |
| NB500882:12:HH75BXX:1:23210:20571:1178 | nuclear protein UL24 [Equid herpesvirus 5] | 9E-17 | 56 | 64 | 192 |
| NB500882:12:HH75BXX:1:21306:3230:2424 | DNA polymerase [Macaca nemestrina rhadinovirus 2] | 3E-25 | 74 | 61 | 183 |
| NB500882:12:HH75BXX:2:12212:14878:11081 | hypothetical protein [Bovine herpesvirus 4] | 9E-17 | 78 | 46 | 138 |
| NB500882:12:HH75BXX:3:12405:22862:2922 | homolog of HVS-2 ORF 25, EBV BcLF1 [Phocid herpesvirus 2] | 1E-16 | 81 | 42 | 126 |
| NB500882:12:HH75BXX:3:23410:25164:4188 | protein UL87 [Equid herpesvirus 5] | 2E-22 | 74 | 58 | 174 |
| NB500882:12:HH75BXX:1:23107:3258:7218 | DNA polymerase [Pan troglodytes rhadinovirus 1] | 3E-21 | 84 | 51 | 153 |
| NB500882:12:HH75BXX:1:12201:14544:19721 | ORF39 [Macaca nemestrina rhadinovirus 2] | 3E-25 | 75 | 71 | 213 |
| NB500882:12:HH75BXX:1:11101:6482:12028 | ORF 68 [Human herpesvirus 8 type M] | 3E-17 | 53 | 62 | 186 |
| NB500882:12:HH75BXX:1:11302:4877:1405 | virion tegument protein [Ateline herpesvirus 3] | 2E-15 | 54 | 70 | 210 |
| NB500882:12:HH75BXX:1:11105:18093:7414 | gB protein [Saimiriine herpesvirus 2] | 6E-27 | 75 | 67 | 201 |
| NB500882:12:HH75BXX:1:21211:4948:5881 | unknown [Porcine lymphotropic herpesvirus 2] | 9E-15 | 56 | 61 | 183 |
| NB500882:12:HH75BXX:1:12306:9801:5799 | virion tegument protein [Ateline herpesvirus 3] | 1E-24 | 78 | 58 | 174 |
| NB500882:12:HH75BXX:2:23307:11552:16910 | tegument protein [Bovine herpesvirus 4] | 5E-21 | 58 | 85 | 255 |
| NB500882:12:HH75BXX:2:23106:4578:12891 | JM43 [Macaca fuscata rhadinovirus] | 8E-22 | 72 | 60 | 180 |
| NB500882:12:HH75BXX:3:12405:6157:16558 | major capsid protein [Bovine herpesvirus 4] | 4E-31 | 78 | 73 | 219 |
| NB500882:12:HH75BXX:3:21609:12030:2870 | DNA replication protein [Macacine herpesvirus 5] | 2E-12 | 48 | 81 | 243 |
| NB500882:12:HH75BXX:3:21508:4328:15204 | glycoprotein H [Ateline herpesvirus 3] | 6E-14 | 61 | 54 | 162 |
| NB500882:12:HH75BXX:3:22507:5067:16992 | transport protein [Ateline herpesvirus 3] | 3E-16 | 54 | 65 | 195 |
| NB500882:12:HH75BXX:3:13404:3689:10980 | single-stranded DNA-binding protein MDBP [Bovine herpesvirus 4] | 1E-19 | 61 | 69 | 207 |
| NB500882:12:HH75BXX:3:23402:22090:15380 | helicase [Ateline herpesvirus 3] | 1E-14 | 57 | 60 | 180 |
| NB500882:12:HH75BXX:3:23605:10898:5171 | large subunit of ribonucleotide reductase [Ateline herpesvirus 3] | 7E-13 | 60 | 47 | 141 |
| NB500882:12:HH75BXX:3:22504:5879:15330 | major DNA-binding protein [Saimiriine herpesvirus 2] | 4E-14 | 78 | 45 | 135 |
| NB500882:12:HH75BXX:3:21505:9508:11591 | tegument protein [Bovine herpesvirus 4] | 2E-18 | 84 | 45 | 135 |
| NB500882:12:HH75BXX:4:11411:21508:18448 | orf 10; similar to Raji LF1 [Ateline herpesvirus 3] | 4E-14 | 47 | 68 | 204 |
| NB500882:12:HH75BXX:4:21510:12992:4017 | orf 37 [Ateline herpesvirus 3] | 2E-13 | 64 | 53 | 159 |
| NB500882:12:HH75BXX:4:22506:10903:12759 | orf 43 [Ateline herpesvirus 3] | 7E-21 | 88 | 51 | 153 |
| NB500882:12:HH75BXX:1:23208:16785:16093 | helicase [Saimiriine herpesvirus 2] | 1E-26 | 77 | 66 | 198 |
|  |  |  |  |  |  |
| ***Diphylla ecaudata herpesvirus SD16*** |  |  |  |  |  |
| NB500882:12:HHG75BGXX:2:11104:21979:1806 | DNA replication protein [Bovine herpesvirus 4] | 8E-09 | 48 | 62 | 186 |
| NB500882:12:HHG75BGXX:3:12606:3543:19015 | ribonucleotide reductase large subunit [Bovine herpesvirus 4] | 5E-09 | 52 | 44 | 132 |
| NB500882:12:HHG75BGXX:1:23201:22457:5121 | DPOL [Human herpesvirus 8] | 2E-11 | 68 | 41 | 123 |
| NB500882:12:HHG75BGXX:4:22603:20590:5012 | thymidylate synthase [Equid herpesvirus 5] | 2E-36 | 84 | 74 | 222 |
| NB500882:12:HHG75BGXX:1:11110:16960:2304 | ORF75 [Ovine herpesvirus 2] | 1E-27 | 60 | 82 | 246 |
| NB500882:12:HHG75BGXX:1:11209:22434:18519 | BTRF1 protein [Human herpesvirus 4] | 2E-13 | 46 | 67 | 201 |
| NB500882:12:HHG75BGXX:1:12109:11237:1125 | ORF6 [Ovine herpesvirus 2] | 3E-17 | 65 | 62 | 186 |
| NB500882:12:HHG75BGXX:1:21203:25245:5634 | terminase [Miniopterus schreibersii rhadinovirus] | 5E-15 | 65 | 55 | 165 |
| NB500882:12:HHG75BGXX:1:23202:15349:1221 | major capsid protein [Harp seal herpesvirus] | 2E-16 | 76 | 51 | 153 |
| NB500882:12:HHG75BGXX:2:11104:24445:12727 | ORF69 [Felis catus gammaherpesvirus 1] | 3E-16 | 54 | 67 | 201 |
| NB500882:12:HHG75BGXX:2:23108:1983:3250 | glycoprotein gM [Macaca mulatta rhadinovirus] | 1E-14 | 71 | 49 | 147 |
| NB500882:12:HHG75BGXX:3:11511:23392:16419 | nuclear egress membrane protein [Equid herpesvirus 5] | 6E-31 | 61 | 90 | 270 |
| NB500882:12:HHG75BGXX:3:13408:16620:14996 | hypothetical protein [Bovine herpesvirus 4] | 1E-18 | 49 | 82 | 246 |
| NB500882:12:HHG75BGXX:3:12511:6920:7076 | BBLF4 [Human herpesvirus 4] | 3E-30 | 78 | 68 | 204 |
| NB500882:12:HHG75BGXX:3:13403:6397:12746 | major capsid protein [Bovine herpesvirus 4] | 5E-22 | 67 | 64 | 192 |
| NB500882:12:HHG75BGXX:3:22411:25405:1806 | putative membrane protein BLRF1 [Human herpesvirus 4] | 6E-12 | 67 | 45 | 135 |
| NB500882:12:HHG75BGXX:4:23607:10200:8164 | ribonucleotide reductase small subunit [Harp seal herpesvirus] | 6E-32 | 65 | 82 | 246 |
| NB500882:12:HHG75BGXX:4:23603:25177:17094 | DNA replication protein [Macacine herpesvirus 5] | 5E-16 | 63 | 52 | 156 |
| NB500882:12:HHG75BGXX:4:22606:17190:2093 | uracil-DNA glycosylase [Equid herpesvirus 2] | 1E-19 | 72 | 53 | 159 |
| NB500882:12:HHG75BGXX:4:11606:14164:3290 | major ssDNA binding protein [Ateline herpesvirus 3] | 5E-12 | 82 | 33 | 100 |
| NB500882:12:HHG75BGXX:1:11211:14518:17303 | helicase-primase subunit [Murid herpesvirus 4] | 6E-19 | 48 | 87 | 261 |
| NB500882:12:HHG75BGXX:2:12307:17115:7379 | orf 39 [Ateline herpesvirus 3] | 1E-22 | 70 | 84 | 252 |
| NB500882:12:HHG75BGXX:2:13203:16462:19054 | ribonucleotide reductase large subunit [Harp seal herpesvirus] | 2E-24 | 70 | 66 | 198 |
| NB500882:12:HHG75BGXX:3:23509:21609:6014 | hypothetical protein [Saimiriine herpesvirus 2] | 6E-09 | 40 | 93 | 279 |
| NB500882:12:HHG75BGXX:3:12409:19644:7224 | hypothetical protein [Bovine herpesvirus 4] | 5E-13 | 40 | 82 | 246 |
| NB500882:12:HHG75BGXX:3:21509:23621:15108 | orf 39 [Ateline herpesvirus 3] | 1E-22 | 70 | 84 | 252 |
| NB500882:12:HHG75BGXX:3:21401:20634:4666 | unknown [Bovine herpesvirus 4] | 2E-23 | 64 | 73 | 219 |
| NB500882:12:HHG75BGXX:3:13505:13374:14395 | DNA polymerase [Phocid herpesvirus 7] | 2E-17 | 69 | 72 | 216 |
| NB500882:12:HHG75BGXX:3:21612:19778:5959 | hypothetical protein [Saimiriine herpesvirus 2] | 7E-12 | 40 | 72 | 216 |
| NB500882:12:HHG75BGXX:3:23507:6748:18116 | orf 41 [Ateline herpesvirus 3] | 2E-13 | 42 | 66 | 198 |
| NB500882:12:HHG75BGXX:3:23510:4327:3794 | unknown protein [Rhesus monkey rhadinovirus H26-95] | 1E-10 | 46 | 61 | 183 |
| NB500882:12:HHG75BGXX:3:13608:6204:6505 | unnamed protein product [Saimiriine herpesvirus 2] | 1E-15 | 69 | 51 | 153 |
| NB500882:12:HHG75BGXX:3:21407:15126:8701 | envelope glycoprotein M [Wood mouse herpesvirus] | 2E-11 | 85 | 34 | 102 |
