# Supplementary material for: Bats, Primates, and the Evolutionary Origins and Diversification of Mammalian Gammaherpesviruses
Source: mBio. 2016 Nov 8;7(6):e01425-16. doi: 10.1128/mBio.01425-16 (PMC5101351; doi:10.1128/mBio.01425-16)
Supplement: Table S4 — BLASTX search results for assembled contigs from the filtered γHVs reads. [file mbo005163037st4.docx]

**TABLE S4** Blastx results for assembled contigs from the filtered γHVs reads

| **CONTIG** | **BLAST ID** | **Target** | **% Cover** | **E-value** | **% ID** | **Lenght (aa)** | **Lenght (nt)** |
| --- | --- | --- | --- | --- | --- | --- | --- |
| CONTIG_1_Desmodus_rotundus_MOR4 | tegument protein/v-FGAM-synthetase | Bovine herpesvirus 4 (NP_076568.1) | 100 | 9E-15 | 40 | 121 | 363 |
| CONTIG_2_Desmodus_rotundus_MOR4 | beta-1,6-N-acetylglucosaminyltransferase | Bovine herpesvirus 4 (NP_076572.1) | 99 | 6E-164 | 92 | 245 | 735 |
|  |  |  |  |  |  |  |  |
| CONTIG_1_Diphylla_ecaudata_SD16 | Bo17, Core-2/I-Branching enzyme | Bovine herpesvirus 4 (AAN18277.1) | 100 | 4E-97 | 69 | 195 | 585 |
| CONTIG_2_Diphylla_ecaudata_SD16 | Orf39, glycoprotein M | Ateline herpesvirus 3 (NP_048012.1) | 100 | 2E-30 | 72 | 94 | 282 |
|  |  |  |  |  |  |  |  |
| CONTIG_1_Diphylla_ecaudata_SD12 | Virion tegument protein aa 155-219 | Ateline herpesvirus 3 (NP_047990.1) | 100 | 2E-31 | 75 | 65 | 195 |
| CONTIG_2_Diphylla_ecaudata_SD12 | Virion tegument protein aa 228-413 | Ateline herpesvirus 3 (NP_047990.1) | 97 | 5E-25 | 59 | 87 | 261 |
