# Supplementary material for: Bats, Primates, and the Evolutionary Origins and Diversification of Mammalian Gammaherpesviruses
Source: mBio. 2016 Nov 8;7(6):e01425-16. doi: 10.1128/mBio.01425-16 (PMC5101351; doi:10.1128/mBio.01425-16)
Supplement: Table S5 — Accession numbers for the DNA polymerase (dpol) and glycoprotein B (gB) sequences from γHVs used in this study. [file mbo005163037st5.docx]

**TABLE S5** Accession numbers for the DNA polymerase (*dpol*) and glycoprotein B (*gB*) sequences from γHVs used in this study

| **DPOL**^1^ | | |  | **gB**^1^ | | |
| --- | --- | --- | --- | --- | --- | --- |
| Name | Accession number | Abbreviation | Common host name | Name | Accession number | Abbreviation |
| Alcelaphine herpesvirus 1 | NC_002531.1 | ALC_HV1 | Wildebeest | Alcelaphine herpesvirus 1 | NC_002531.1 | ALC_HV1 |
| Apodemus sylvaticus rhadinovirus 1 | EF128051.2 | APSY_RHV1 | Wood mouse | Apodemus sylvaticus rhadinovirus 1 | EF128051.2 | APSY_RHV1 |
| Ateline herpesvirus 3 | NC_001987.1 | ATE_HV3 | Woolly monkey | Ateline herpesvirus 3 | AF083424.1 | ATE_HV3 |
| Babyrousa babyrussa rhadinovirus 1 | AY177146.2 | BABA_RHV1 | Golden babirusa hog | Babyrousa babyrussa rhadinovirus 1 | AY177146.2 | BABA_RHV1 |
| Bandicota indica rhadinovirus 4 | EF128043.1 | BAIN_RHV4 | Greater bandicoot rat | Bandicota indica rhadinovirus 4 | EF128043.1 | BAIN_RHV4 |
| Bandicota savilei rhadinovirus 1 | DQ821581.1 | BASA_RHV1 | Savile's bandicoot rat | Bandicota savilei rhadinovirus 1 | DQ821581.1 | BASA_RHV1 |
| Bovine herpesvirus 4 | AF318573.1 | BOS_HV4 | Cattle | Bovine herpesvirus 4 | AF318573.1 | BOS_HV4 |
| Bovine herpesvirus 6 | NC_024303.1 | BOS_HV6 | Cattle | Bovine herpesvirus 6 | KJ705001.1 | BOS_HV6 |
| Bovine lymphotropic herpesvirus | AF327830.1 | BOS_LHV | Cattle | Bovine lymphotropic herpesvirus | AF327832.1 | BOS_LHV |
| Callitrichine herpesvirus 3 | AF319782.2 | CAL_HV3 | Marmoset | Callitrichine herpesvirus 3 | AF319782.2 | CAL_HV3 |
| Caprine herpesvirus 2 | HQ116812.1 | CAP_HV2 | Goat | Caprine herpesvirus 2 | AF283477.2 | CAP_HV2 |
| Crocuta crocuta gammaherpesvirus 1 | DQ789371.2 | CRO_GHV1 | Spotted hyena | Crocuta crocuta gammaherpesvirus 1 | DQ789371.2 | CRO_GHV1 |
| *Cynopterus sphinx CS/12GZ1* | KR261850 | CYSP_M102 | Greater short-nosed fruit bat | *Cynopterus sphinx 13HN70* | KR261906 | CYSP_70 |
| *Cynopterus sphinx CS/14GZ24* | KR261898 | CYSP_M24 | Greater short-nosed fruit bat |  |  |  |
| Diceros bicornis gammaherpesvirus 1 | AY197560.2 | DIBI_GHV | Black rhinoceros | Diceros bicornis gammaherpesvirus | AY197560.2 | DIEC_SD12 |
| Elephas maximus gammaherpesvirus 1 | EU085379.1 | ELMA_GHV1 | Asian elephant | Elephas maximus gammaherpesvirus 1 | EU085379.1 | ELMA_GHV1 |
| *Eptesicus serotinus rhadinovirus 1* | DQ788623.2 | EPSE_RHV1 | Serotine bat | *Eptesicus serotinus rhadinovirus 1* | DQ788623.2 | EPSE_RHV1 |
| Equid herpesvirus 2 | U20824.2 | EQ_HV2 | Horse | Equid herpesvirus 2 | U20824.2 | EQ_HV2 |
| Equid herpesvirus 5 | KM924295.1 | EQ_HV5 | Horse | Equid herpesvirus 5 | KM924295.1 | EQ_HV5 |
| Equus zebra gammaherpesvirus 1 | AY495965.2 | EQZ_GHV1 | Zebra | Equus zebra gammaherpesvirus 1 | AY495965.2 | EQZ_GHV1 |
| Felis catus gammaherpesvirus 1 | NC_028099.1 | FECA_GHV1 | Cat | Felis catus gammaherpesvirus 1 | NC_028099.1 | FECA_GHV1 |
| Gorilla gorilla lymphocryptovirus 1 | AF534225.3 | GOGO_LCV | Gorilla | Gorilla gorilla lymphocryptovirus 1 | AF534225.3 | GOGO_LCV |
| Gorilla rhadinovirus 1 | AY177144.2 | GOGO_RHV1 | Gorilla | Gorilla rhadinovirus 1 | AY177144.2 | GOGO_RHV1 |
| Hexaprotodon liberiensis gammaherpesvirus 1 | AY197559.2 | HELI_GHV1 | Pygmy hippopotamus | Hexaprotodon liberiensis gammaherpesvirus 1 | AY197559.2 | HELI_GHV1 |
| *Hipposideros diadema herpesvirus* | AB490083.2 | HIDI_HV | Diadem leaf-nosed bat | *Hipposideros diadema herpesvirus* | AB490083.2 | HIDI_HV |
| *Hipposideros larvatus HL/11HN1* | KR261841 | HL_HN1 | Intermediate roundleaf bat |  |  |  |
| *Hipposideros pomona HP/11HN104* | KR261843 | HP_104 | Pomona roundleaf bat | *Hipposideros pomona 211HN104* | KR261902 | HP_104 |
| *Hipposideros pomona HP/11HN110* | KR261845 | HP_110 | Pomona roundleaf bat |  |  |  |
| Human herpesvirus 4 | LN831023.1 | HS_HV4 | Human | Human herpesvirus 4 | NC_007605.1 | HS_HV4 |
| Human herpesvirus 8 | AF005477.2 | HS_HV8 | Human | Human herpesvirus 8 | AF092928.1 | HS_HV8 |
| Lynx rufus gammaherpesvirus 1 | KF840716.1 | LYRU_GHV1 | Bobcat | Lynx rufus gammaherpesvirus 1 | KF840716.1 | LYRU_GHV1 |
| Macaca fascicularis lymphocryptovirus 1 | AF534221.2 | MAFA_LCV | Crab-eating macaque | Macaca fascicularis lymphocryptovirus 1 | AF534221.2 | MAFA_LCV |
| Macaca fascicularis rhadinovirus 2 | EU085377.1 | MAFA_RHV2 | Crab-eating macaque | Macaca fascicularis rhadinovirus 2 | EU085377.1 | MAFA_RHV2 |
| Macaca fuscata rhadinovirus | AY528864.1 | MAFU_RHV | Japanese macaque | Macaca fuscata rhadinovirus | AY528864.1 | MAFU_RHV |
| *Miniopterus schreibersii MS/11HN95* | KR261846 | MSC_95 | Common bent-wing bat | *Miniopterus schreibersii 11HN110* | KR261903 | MSC_110 |
| *Miniopterus schreibersii MS/12HN28* | KR261851 | MSC_28 | Common bent-wing bat | *Miniopterus schreibersii 211HN16* | KR261901 | MSC_16 |
| Murid herpesvirus 4 | NC_001826.2 | MUR_HV4 | Mouse | Murid herpesvirus 4 | NC_001826.2 | MUR_HV4 |
| Mus cervicolor rhadinovirus 1 | DQ821582.1 | MUCE_RHV1 | Fawn-colored mouse | Mus cervicolor rhadinovirus 1 | DQ821582.1 | MUCE_RHV1 |
| Mus musculus rhadinovirus 1 | AY854167.1 | MUMUS_RHV1 | House mouse | Mus musculus rhadinovirus 1 | AY854167.1 | MUMUS_RHV1 |
| Mustelid herpesvirus 1 | AF376034.1 | MUST_HV1 | Ferret | Mustelid herpesvirus 1 | AF376034.1 | MUST_HV1 |
| Myodes glareolus rhadinovirus 1 | AY854169.2 | MYGLA_RHV1 | Bank vole | Myodes glareolus rhadinovirus 1 | AY854169.2 | MYGLA_RHV1 |
| *Myotis nattereri rhadinovirus 1* | DQ788625.1 | MYNA_RHV1 | Natterer's bat |  |  |  |
| *Myotis ricketti herpesvirus 1* | JN692429.1 | MYRI_HV1 | Rickett's big-footed bat | *Myotis ricketti herpesvirus 1* | JN692429.1 | MYRI_HV1 |
| *Myotis ricketti herpesvirus 2* | JN692430.1 | MYRI_HV2 | Rickett's big-footed bat | *Myotis ricketti herpesvirus 2* | JN692430.1 | MYRI_HV2 |
| *Myotis velifer gammaherpesvirus 8* | KU220026.1 | MYVE_HV8 | Cave myotis | *Myotis velifer gammaherpesvirus 8* | KU220026.1 | MYVE_HV8 |
| *Nyctalus noctula rhadinovirus 1* | DQ788626.1 | NYNOC_RHV1 | Common noctule bat |  |  |  |
| *Nyctalus noctula rhadinovirus 2* | DQ788627.2 | NYNOC_RHV2 | Common noctule bat | *Nyctalus noctula rhadinovirus 2* | DQ788627.2 | NYNOC_RHV2 |
| Ovine herpesvirus 2 | NC_007646.1 | OVI_HV2 | Sheep | Ovine herpesvirus 2 | NC_007646.1 | OVI_HV2 |
| Pan troglodytes rhadinovirus 2 | EU118145.1 | PATR_RHV2 | Common chimpanzee | Pan troglodytes rhadinovirus 2 | EU085378.1 | PATR_RHV2 |
| Pan troglodytes rhadinovirus 3 | GQ995451.1 | PATR_RHV3 | Common chimpanzee | Pan troglodytes rhadinovirus 3 | GQ995451.1 | PATR_RHV3 |
| Panthera leo gammaherpesvirus 1 | DQ789370.2 | PALEO_GHV1 | Lion | Panthera leo gammaherpesvirus 1 | DQ789370.2 | PALEO_GHV1 |
| Papio hamadryas lymphocryptovirus 2 | AF534229.3 | PAHAM_LCV2 | Hamadryas baboon | Papio hamadryas lymphocryptovirus 2 | AF534229.3 | PAHAM_LCV2 |
|  |  |  | Koala |  |  |  |
| *Pipistrellus nathusii rhadinovirus 1* | DQ788629.2 | PIPNA_RHV1 | Nathusius's pipistrelle bat | *Pipistrellus nathusii rhadinovirus 1* | DQ788629.2 | PIPNA_RHV1 |
| *Pipistrellus pipistrellus rhadinovirus 1* | DQ788630.1 | PIPI_RHV1 | Common pipistrelle bat |  |  |  |
| *Plecotus auritus rhadinovirus 1* | DQ788628.1 | PLAUR_RHV1 | Brown long-eared bat |  |  |  |
|  |  |  |  | Porcine lymphotropic herpesvirus 1 | AF478169.1 | SUS_LTV1 |
| Porcine lymphotropic herpesvirus 2 | AF191043.1 | SUS_LTV2 | Pig | Porcine lymphotropic herpesvirus 2 | AY170317.1 | SUS_LTV2 |
| Porcine lymphotropic herpesvirus 3 | AY170316.1 | SUS_LTV3 | Pig | Porcine lymphotropic herpesvirus 3 | AY170316.1 | SUS_LTV3 |
| Procavia capensis gammaherpesvirus 2 | JF705865.1 | PROCA_GHV2 | Rock hyrax | Procavia capensis gammaherpesvirus 2 | JF705865.1 | PROCA_GHV2 |
| *Ptenochirus jagori gammaherpesvirus* | LC008326.1 | PTEJA_GHV | Greater musky fruit bat |  |  |  |
| *Pteropus giganteus herpesvirus 1* | KC692446.1 | PTGIG_HV1 | Indian flying fox |  |  |  |
| *Pteropus giganteus herpesvirus 2* | KC692449.1 | PTGIG_HV2 | Indian flying fox |  |  |  |
| *Pteropus giganteus herpesvirus 3* | KC692450.1 | PTGIG_HV3 | Indian flying fox |  |  |  |
| *Pteropus giganteus herpesvirus 5* | KC692447.1 | PTGIG_HV5 | Indian flying fox |  |  |  |
| *Pteropus giganteus herpesvirus 6* | KC692448.1 | PTGIG_HV6 | Indian flying fox |  |  |  |
| Puma concolor gammaherpesvirus 1 | KF840717.1 | PUCON_GHV1 | Cougar | Puma concolor gammaherpesvirus 1 | KF840717.1 | PUCON_GHV1 |
| *Rhinolophus blythi RB/13YF11* | KR261856 | RHIBLY_F11 | Blyth's horsehoe bat | *Rhinolophus blythi 13HN56* | KR261905 | RHIBLY_56 |
| *Rhinolophus blythi RB/13YF3* | KR261882 | RHIBLY_YF3 | Blyth's horsehoe bat | *Rhinolophus blythi 13YF104* | KR261913 | RHIBLY_104 |
| *Rhinolophus blythi RB/13YF6* | KR261883 | RHIBLY_YF6 | Blyth's horsehoe bat | *Rhinolophus blythi 13YF79* | KR261908 | RHIBLY_79 |
| *Rhinolophus blythi RB/13YF84* | KR261887 | RHIBLY_F84 | Blyth's horsehoe bat | *Rhinolophus blythi 13YF82* | KR261910 | RHIBLY_F82 |
| *Rhinolophus blythi RB/13YF87* | KR261889 | RHIBLY_F87 | Blyth's horsehoe bat | *Rhinolophus blythi 13YF84* | KR261909 | RHIBLY_F84 |
| *Rhinolophus blythi RB/13YF89* | KR261891 | RHIBLY_F89 | Blyth's horsehoe bat | *Rhinolophus blythi 13YF87* | KR261911 | RHIBLY_F87 |
| *Rhinolophus blythi RB/13YF96* | KR261893 | RHIBLY_F96 | Blyth's horsehoe bat | *Rhinolophus blythi 13YF96* | KR261912 | RHIBLY_F96 |
| *Rhinolophus blythi RB/13YF99* | KR261895 | RHIBLY_F99 | Blyth's horsehoe bat |  |  |  |
| Rupicapra rupicapra gammaherpesvirus 1 | DQ789369.2 | RURUP_GHV1 | Chamois | Rupicapra rupicapra gammaherpesvirus 1 | DQ789369.2 | RURUP_GHV1 |
| Saimiri sciureus gammaherpesvirus 2 | AY138584.2 | SAMCI_GHV2 | Common squirrel monkey | Saimiri sciureus gammaherpesvirus 2 | AY138584.2 | SAMCI_GHV2 |
| Saimiriine herpesvirus 2 | NC_001350.1 | SAM_HV2 | Common squirrel monkey | *Scotophilus kuhlii 11HZ76* | KR261904 | SCKUH_76 |
| *Scotophilus kuhlii SK/11HZ84* | KR261848 | SCKUH_84 | Lesser Asiatic yellow bat | *Scotophilus kuhlii 13Y234* | KR261920 | SCKUH_234 |
| *Scotophilus kuhlii SK/13YF121* | KR261858 | SCKUH_M121 | Lesser Asiatic yellow bat | *Scotophilus kuhlii 13YF106* | KR261914 | SCKUH_106 |
| *Scotophilus kuhlii SK/13YF14* | KR261863 | SCKUH_14 | Lesser Asiatic yellow bat | *Scotophilus kuhlii 13YF114* | KR261915 | SCKUH_114 |
| *Scotophilus kuhlii SK/13YF146* | KR261865 | SCKUH_146 | Lesser Asiatic yellow bat | *Scotophilus kuhlii 13YF15* | KR261907 | SCKUH_15 |
| *Scotophilus kuhlii SK/13YF15* | KR261866 | SCKUH_15 | Lesser Asiatic yellow bat | *Scotophilus kuhlii 13YF155* | KR261916 | SCKUH_155 |
| *Scotophilus kuhlii SK/13YF16* | KR261872 | SCKUH_16 | Lesser Asiatic yellow bat | *Scotophilus kuhlii 13YF160* | KR261917 | SCKUH_160 |
| *Scotophilus kuhlii SK/13YF185* | KR261876 | SCKUH_M185 | Lesser Asiatic yellow bat | *Scotophilus kuhlii 13YF187* | KR261918 | SCKUH_187 |
| *Scotophilus kuhlii SK/13YF239* | KR261880 | SCKUH_239 | Lesser Asiatic yellow bat | *Scotophilus kuhlii 13YF206* | KR261919 | SCKUH_206 |
|  |  |  |  | *Scotophilus kuhlii 13YF244* | KR261921 | SCKUH_244 |
| Sorex araneus gammaherpesvirus 1 | EU085380.1 | SORA_GHV1 | Common shrew | Sorex araneus gammaherpesvirus 1 | EU085380.1 | SORA_GHV1 |
| Sus barbatus rhadinovirus 1 | AY177147.2 | SUSBA_RHV1 | Bornean bearded pig | Sus barbatus rhadinovirus 1 | AY177147.2 | SUSBA_RHV1 |
| Symphalangus syndactylus lymphocryptovirus 2 | GQ921924.1 | SYMSY_LCV2 | Siamang gibbon | Symphalangus syndactylus lymphocryptovirus 2 | GQ921924.1 | SYMSY_LCV2 |
| Tapirus terrestris gammaherpesvirus 1 | AF141887.3 | TATER_GHV1 | South American tapir | Tapirus terrestris gammaherpesvirus 1 | AF141887.3 | TATER_GHV1 |
| Tupaia belangeri gammaherpesvirus 1 | AY197561.2 | TUBEL_GHV1 | Northern treeshrew | Tupaia belangeri gammaherpesvirus 1 | AY197561.2 | TUBEL_GHV1 |
| Type 2 ruminant rhadinovirus of mule deer | HM014314.1 | T2MDRHV | Mule deer | Type 2 ruminant rhadinovirus of mule deer | HM014314.1 | T2MDRHV |
|  |  |  |  |  |  |  |
| Suid alphaherpesvirus 1 | JQ809330.1 | SUS_AHV1 | Pig | Suid alphaherpesvirus 1 | NC_006151.1 | SUS_AHV1 |
| Human herpes simplex virus type 1 | M10792.1 | HS_AHV1 | Human | Human herpes simplex virus type 1 | JN555585.1 | HS_AHV1 |
| *Pteropus dasymallus alphaherpesvirus 1* | NC_024306.1 | PTEDA_AHV1 | Ryukyu flying fox | *Pteropus dasymallus alphaherpesvirus 1* | AB825953.1 | PTEDA_AHV1 |
| *Cynopterus sphinx betaherpesvirus CS/14GZ13* | KR261896 | CYSP_A13 | Greater short-nosed fruit bat |  |  |  |
| *Cynopterus sphinx betaherpesvirus CS/14GZ9* | *KR261900* | CYSP_A9 | Greater short-nosed fruit bat |  |  |  |

^1^ Bat sequences are shown in cursive letters
