# Supplementary material for: Bats, Primates, and the Evolutionary Origins and Diversification of Mammalian Gammaherpesviruses
Source: mBio. 2016 Nov 8;7(6):e01425-16. doi: 10.1128/mBio.01425-16 (PMC5101351; doi:10.1128/mBio.01425-16)
Supplement: Table S6 — Significant identities determined by PSI-BLAST between the amino acid sequences of the viral and host FLIP/FLAR proteins. [file mbo005163037st6.docx]

**TABLE S6** Significant identities determined by PSI-BLAST between the amino acid sequences of the viral and host FLIP/FLAR proteins.

| **Description**^1^ | **Query cover** | **E value** | **% Identity** | **Accession** | **Common Name** |
| --- | --- | --- | --- | --- | --- |
| vFLIP [Bovine herpesvirus 4] | 100% | 5E-130 | 100% | AIA82816.1 |  |
| cFLAR [Aotus nancymaae] | 94% | 2E-22 | 35% | XP_012301599.1 | Night monkey |
| cFLAR [Callithrix jacchus] | 94% | 5E-22 | 34% | XP_008997452.1 | Marmoset |
| cFLAR [Rattus norvegicus] | 93% | 8E-22 | 32% | NP_476479.1 | Rat |
| cFLAR [Sus scrofa] | 94% | 8E-22 | 34% | AAS22336.1 | Pig |
| cFLAR [Galeopterus variegatus] | 95% | 1E-21 | 32% | XP_008562088 | Flying lemur |
|  |  |  |  |  |  |
| vFLIP [Equid herpesvirus 2] | 100% | 1E-121 | 100% | NP_042671.1 |  |
| cFLAR [Nannospalax galili] | 93% | 6E-19 | 39% | XP_008837000.1 | Mole-rat |
| cFLAR [Capra hircus] | 93% | 2E-16 | 36% | ABQ57332.1 | Goat |
| cFLAR [Ovis aries] | 93% | 6E-15 | 36% | XP_004004869.1 | Sheep |
|  |  |  |  |  |  |
| vFLIP [Equid herpesvirus 5] | 100% | 1E-123 | 100% | YP_009118464.1 |  |
| cFLAR [Nannospalax galili] | 100% | 1E-18 | 35% | XP_008836999.1 | Mole-rat |
| cFLAR [Capra hircus] | 100% | 1E-17 | 33% | ABQ57332.1 | Goat |
| cFLAR [Carlito syrichta] | 100% | 4E-17 | 33% | XP_008068976.1 | Tarsier |
| cFLAR [Myotis davidii] | 100% | 2E-16 | 34% | XP_015414634.1 | David's myotis |
|  |  |  |  |  |  |
| vFLIP [Felis catus gammaherpesvirus 1] | 100% | 2E-131 | 100% | YP_009173878.1 |  |
| cFLAR [Ursus maritimus] | 87% | 1E-05 | 29% | XP_008685572.1 | Polar bear |
|  |  |  |  |  |  |
| vFLIP/K13 [Human herpesvirus 8] | 100% | 4E-134 | 100% | YP_001129429.1 |  |
| cFLAR [Dipodomys ordii] | 86% | 2E-22 | 37% | XP_012867414.1 | Kangaroo rat |
| cFLAR [Myotis lucifugus] | 87% | 1E-19 | 34% | XP_014316918.1 | Little brown bat |
| cFLAR [Myotis davidii] | 87% | 2E-19 | 34% | XP_015414634.1 | David's myotis |
| cFLAR [Myotis brandtii] | 87% | 2E-18 | 33% | XP_014401490.1 | Brandt's bat |
|  |  |  |  |  |  |
| vFLIP [Macaca fuscata rhadinovirus] | 100% | 6E-110 | 100% | AAT00128.1 |  |
| cFLAR [Nannospalax galili] | 96% | 2E-09 | 26% | XP_017655054.1 | Mole-rat |
| cFLAR [Dipodomys ordii] | 96% | 3E-09 | 27% | XP_012867414.1 | Kangaroo rat |
| cFLAR [Cebus capucinus imitator] | 96% | 3E-09 | 28% | XP_017400766.1 | White-headed monkey |
|  |  |  |  |  |  |
| vFLIP [Myotis gammaherpesvirus 8] | 71% | 1E-134 | 100% | YP_009229831.1 |  |
| cFLAR [Myotis lucifugus] | 94% | 2E-71 | 50% | XP_014316918.1 | Little brown bat |
| cFLAR [Myotis davidii] | 94% | 1E-70 | 50% | XP_015414634.1 | David's myotis |
| cFLAR [Myotis brandtii] | 94% | 3E-70 | 50% | XP_014401490.1 | Brandt's bat |
| cFLAR [Sus scrofa] | 94% | 1E-64 | 46% | AAS22336.1 | Pig |

^1^ For ATE_HV3, MAFU_RHV and SAM_HV2 no homology to mammalian proteins was detected
